# Nature of innovations affecting photovoltaic system costs

**DOI:** 10.1371/journal.pone.0320676

**Published:** 2025-08-11

**Authors:** Goksin Kavlak, Magdalena M. Klemun, Ajinkya S. Kamat, Brittany L. Smith, Robert M. Margolis, Jessika E. Trancik

**Affiliations:** 1 Institute for Data, Systems and Society, Massachusetts Institute of Technology, Cambridge, Massachusetts, United States of America; 2 Department of Civil and Systems Engineering, Johns Hopkins University, Baltimore, Maryland, United States of America; 3 National Renewable Energy Laboratory, Washington, DC, United States of America; 4 Santa Fe Institute, Santa Fe, New Mexico, United States of America; University of Oxford, UNITED KINGDOM OF GREAT BRITAIN AND NORTHERN IRELAND

## Abstract

Innovations improve technology costs through various kinds of engineering advancements, including changes to materials choices and device or process designs. Understanding how these innovations relate to cost change can reveal aspects of the process of technology evolution, yet developing such understanding is often not possible with a strictly quantitative approach due to data limitations. In this paper we develop a hybrid quantitative-qualitative framework for relating specific innovations to cost change by using the variables in a quantitative technology cost change model as an organizing principle. We demonstrate this framework by applying it to the cost decline in photovoltaic (PV) systems over the last five decades. This framework generates new understanding of a set of innovations that contributed to PV modules’ sustained cost decline and the more modest trends observed in balance-of-system (BOS) costs. The results show the great diversity of innovations that affected PV costs, drawing on wide-ranging fields of expertise within scientific research and practice. We find that there are differences in the characteristics of innovations that reduced the cost of PV modules compared to innovations influencing BOS costs. Numerous module innovations reduced costs by advancing manufacturing tools and processes that improved material quality. Many BOS innovations reduced costs through a combination of component design changes, integration, automation, digitalization, and standardization. Overall, most innovations in our sample affected PV hardware. However, some also target ‘soft technologies’ such as task durations through innovations like fast-track permitting, which require improved collaboration and process streamlining. This framework also provides insight into the nature of knowledge spillovers between technologies. Both module and BOS hardware innovations show the benefits of PV’s position within an ‘ecosystem’ of continuously advancing technologies in many industries, in particular semiconductors and electronics, and also point to the importance of public institutions for accelerating testing, permitting, and training.

## 1 Introduction

Energy technologies such as photovoltaics (PV) modules and wind turbines have declined rapidly in cost over the last five decades [1–3], and previous literature suggests that innovation activity has been one of the most important drivers of this rapid cost reduction [4–6]. While many studies have focused on identifying the cost effects of innovations at an aggregate level as represented by R&D investments and patenting [2, 4], less emphasis has been placed on connecting specific innovations to technology costs. Revealing the specific innovations leading to cost decline can contribute important understanding of the reasons for observed improvement in a particular technology and the process of technology improvement more generally.

In this paper, we advance a hybrid framework combining qualitative and quantitative methods to characterize the innovations that affect costs. We apply this framework to innovations affecting the cost of crystalline silicon PV and ask: Which specific innovations have influenced technology features and cost components of PV systems in the past? Are there differences in innovation patterns between modules and balance-of-system (BOS), the two components of PV systems?

A common approach to studying the relationship between innovation and technology costs has been experience curve modeling (e.g., [7]). Experience curves show the correlation between the cost of a technology and the level of effort put into developing, improving, and using the technology, which can be represented with proxies such as cumulative deployment or, less commonly, R&D investment, and patents [2, 8]. One-factor experience curves measure the correlation between cost and yearly or cumulative deployment, which is an aggregate variable combining the effects of scale, learning, and innovations [1]. Multi-factor experience curves have been proposed in order to separate these multiple effects, especially the effect of innovations, by introducing other explanatory variables [4, 9, 10]. These correlation-based models are useful for estimating and predicting the rates of improvement for a range of technologies. They do not, however, explain how innovations and other factors lead to cost changes. A better understanding of the link between innovations, changing technology characteristics, and costs can inform the assessment of past R&D efforts and the design of future efforts.

Innovations reduce cost by improving specific technology performance variables. To connect specific innovations to the cost of a technology, it is, therefore, useful to start from a model that computes cost from a set of measurable variables such as material usage, efficiency, task durations, and labor rates. An innovation can then be described as affecting technology costs if it changes one or more of the variables. This detailed, bottom-up modeling of cost provides an organizing framework for studying how innovations affect technology costs.

In this paper, we advance a new framework with a bottom-up approach to characterize innovations and other factors affecting technology costs. At the center of this framework is a previously developed cost equation for PV systems [11], which shows how different material and labor inputs determine the costs of PV modules and balance-of-systems [11, 12]. By breaking down PV’s cost into variables, this cost equation guides the qualitative study of innovations at detailed levels that are not easily measurable due to the lack of quantitative data. These innovations affected the cost of specific materials, manufacturing steps, and deployment processes. We then develop a typology for the innovations identified in order to better characterize the sources of technological change. The typology classifies each innovation according to how it changed the variables in the cost model, including improvement processes such as automation, standardization, and digitalization. We also investigate when and in which industry individual innovations originated to better understand the nature of knowledge spillovers between technologies. Besides these qualitative approaches to characterizing innovations , we also outline quantitative approaches for estimating the cost impacts of innovations where data can be obtained to populate a cost equation at the level of detail required.

To demonstrate the framework, we apply it to PV innovations. Here, we use the PV systems cost model from [11] to identify the innovations that have affected residential PV system costs since the 1970s. Although this study and the previous work in [11] start with the same cost model, they perform different analyses. Previous work [11, 12] provides quantitative estimates of the contribution of the cost variables, without diving into the specific innovations that operate at one level of further disaggregation and greater detail than the cost variables. This study expands on [11, 12] by providing a qualitative analysis of how specific innovations impact each variable of the cost model.

Based on the resulting table of cost variables, innovations, and innovation types and origins (‘innovations table’), we draw conclusions about the sources of technological change in PV modules and BOS. We also learn valuable lessons about the challenges in arriving at a full suite of innovations due to data limitations, and we propose ways to overcome these limitation in future work. As more data becomes available to track detailed innovations, they can be studied quantitatively by writing out a cost equation with more fine-grained variables capturing the effects of detailed innovations.

Previous research on PV innovations has largely focused on PV modules [12–15]. BOS costs at individual points in time have been modeled using a bottom-up approach [16–18], and some recent papers have used experience curve models to study changing BOS and PV system costs [19–21]. Following the rapid decline of module costs, more focus has been put on soft costs, which have declined less rapidly than hardware costs [18, 22], and vary across countries [16, 17] and regions [18].

In this paper, we examine innovations at both module and BOS levels. Unlike modules, which are mass-produced goods, many BOS components are custom-built and site-specific (e.g., mounting systems). Cost variables associated with BOS, such as installation time and labor rates, describe costs that are not hardwired into the technology and depend on local actors and site-specific conditions [11]. By examining the innovations at the BOS level as well as in modules, we begin to uncover the different innovation types that have been prevalent in these two components of PV technology.

Several studies have investigated innovation in the PV domain by analyzing patents, which are widely used as a proxy for innovative activity [5, 23–25]. These studies have found that PV differs from other energy technologies in (a) the focus of innovative activity, with important PV patents representing both component- and system-level inventions [25]; (b) the temporal evolution of innovative activity, which shows a shift from product innovations to manufacturing process innovations, as common for mass-produced goods but not for ‘complex products’ [5]. Studies on the role of knowledge spillovers between PV and other industries have led to various results. Cross-technology knowledge flows have been identified in some research as important for wind but not as important for PV [26], while other research has pointed to significant external knowledge flows contributing to silicon crystal and epitaxy growth processes, stock materials, and coating processes [27].

The typical distinction drawn between inventions and innovations is that innovations are successful commercial implementations of inventions, while inventions are ideas or technical advances that were deemed novel relative to prior art, but may not be widely adopted or even implemented at all [28, 29]. While previous work has focused primarily on patents, this paper focuses on innovations that affected at least one variable in the PV system cost equation and thus likely influenced PV costs. By choosing innovations as the unit of analysis, our work captures a broad set of improvement activities contributing to PV system cost change over time, since not all efforts generating novel approaches to build and install PV systems may have led to patents. A broader perspective is also needed to compare module to BOS innovations, as many installation-related variables are affected by site- or region-specific processes and, to a lesser degree, by patented innovations.

Our hybrid approach combines a quantitative cost model and qualitative research on innovations in order to provide a tool for linking specific innovations to cost reduction levers. The use of a cost model guides the qualitative research and enables sifting through a rich history of innovations. The application of this hybrid approach contributes insights into knowledge spillovers across technologies and the differences between module and BOS innovations.

This paper is organized as follows. Section 2 describes the method we use to identify and classify a set of innovations, and to collect evidence on their origins and time periods of emergence. Section 3 presents the results obtained from this analysis and then demonstrates approaches to quantifying the cost impacts of innovations. Section 4 discusses the insights gained and the implications for future innovations.

## 2 Methods

### 2.1 Cost model

In this framework, we connect specific innovations to the key determinants of technology costs established by a cost equation. The role of the cost equation in our method is (a) to provide a starting point for a focused literature search, using variable names and synonyms to identify relevant journal papers and technical reports; and (b) to constrain the set of innovations we consider by emphasizing innovations with direct effects on the variables in the cost equation. In our previous work, we developed a method of representing the cost and cost change determinants of a technology in cost and cost change models [11, 12]. Variables in the cost model jointly determine the cost of the technology, and each variable can be influenced by multiple innovations.

Equation 1 gives the cost of a PV system to the system owner before applying any incentives or subsidies [11]. The first factor in the equation accounts for overhead and profit (*p*_*op*_), and converts DC power to AC power by accounting for the inverter output and its conversion efficiency. The first term of the equation represents the cost components of PV modules: silicon costs, other material costs, and an aggregate component for capital, labor, electricity, O&M costs for module manufacturing, and a module factory margin. The following terms represent inverter prices, aluminum costs for the racking system, wire prices, installation costs (electrical and mechanical), design costs, permitting costs, the costs of manufacturing the racking system, as well as the price of electrical hardware needed in addition to the inverter, fees for permitting, inspection and interconnection (PII) activities, and supply chain and sales tax-related expenses.

This equation reflects the system boundaries within which we study cost change processes in depth. Here we draw boundaries around both module manufacturing and PV system installation processes to more closely examine innovations affecting module and BOS costs. (We also examine several key innovations affecting inverter manufacturing costs, though we do not write out an explicit cost equation for inverters.) Also note that while this equation has been written for PV systems, a cost equation can be written for any technology to identify innovations affecting each variable, assuming prior engineering knowledge of cost determinants and the relationships among them.

$$C_{sys}\left(\frac{\$}{W_{ac}}\right)=\frac{1+p_{op}}{K_{inv}\eta_{inv}}[\underset{\text{Module price}}{\underbrace{p_{M}K_{s}}}+\underset{\text{Inverter price}}{\underbrace{p_{inv}K_{s}}}+\frac{1}{\eta_{w}}\;(\underset{\text{racking aluminum costs}}{\underbrace{K_{s}\phi_{a}p_{a}}}+\underset{\text{total wire price}}{\underbrace{\frac{K_{s}\alpha }{A\eta_{m}n_{mc}\sigma }\phi_{w}p_{w}}}+\underset{\text{system design costs}}{\underbrace{\tau_{s}w_{s}}}\;+\underset{\text{mechanical and electrical installation costs}}{\underbrace{\frac{K_{s}\alpha }{A\eta_{m}n_{mc}\sigma }\sum_{i=1}^{2}\tau_{i}w_{i}}}+\underset{\text{PII labor costs}}{\underbrace{\tau_{PII}w_{PII}}}\;+\underset{\text{residual racking price}}{\underbrace{p_{r}}}+\underset{\text{other el. hardware price}}{\underbrace{p_{oe}}})]\;+\frac{1}{K_{inv}\eta_{inv}}\left(\underset{\text{PII fees}}{\underbrace{c_{PII}}}+\underset{\text{supply chain costs}}{\underbrace{c_{sc}}}+\underset{\text{sales tax expenses}}{\underbrace{c_{stax}}}\right),$$ (1)

where the total system price is written as the sum of module price (*p*_*M*_) and BOS costs (see Table 1 for definitions of variables). The module price is the sum of module costs and a margin incurred by the module factory owner. Module costs are modeled as the sum of silicon costs, non-silicon material costs, and plant-size dependent costs (see Table 2, based on [12]), plus a margin charged by the module manufacturer:

$$p_{M}\left(\frac{\$}{W}\right)=\frac{\alpha }{\sigma A\eta_{m}y}\left[Av\rho p_{s}+cA+p_{0}{\left(\frac{K}{K_{0}}\right)}^{-b}\right]+p_{mf}.$$ (2)

**Table 1:** Variables in the cost equation for PV systems (Eq 1).

Symbol	Meaning	Unit
*p*_*op*_	Overhead/profit margin (%)	unitless
$K_{inv}$	Inverter ac power output	*W*_*ac*_
$\eta_{inv}$	Inverter ac efficiency	unitless
*p*_*M*_	Module price	2017$/W_*dc*_
*K*_*s*_	System power	W_*dc*_
$p_{inv}$	Inverter price	2017$/W_*dc*_
$\eta_{w}$	Wiring efficiency	unitless
$\phi_{a}$	Specific aluminum use	kg/W_*dc*_
*p*_*a*_	Aluminum price	2017$/kg
$\phi_{w}$	Wire use	m/module
*p*_*w*_	Wire price	2017$/m
$\tau_{s}$	System design time	h/system
*w*_*s*_	System design wage	2017$/h
*α*	Area utilization	unitless
*A*	Wafer area	m^2^
$\eta_{m}$	Module efficiency	unitless
*n*_*mc*_	Number of cells per module	unitless
*σ*	Solar constant	W_*dc*_/ m^2^
$\tau_{m}$	Mechanical installation time	h/module
*w*_*m*_	Mechanical labor wage	2017$/h
$\tau_{e}$	Electrical installation time	h/module
*w*_*e*_	Electrical labor wage	2017$/h
$\tau_{PII}$	Permitting, inspection, and	h/system
	interconnection (PII) time	
*w*_*PII*_	PII wage	2017$/h
*p*_*r*_	Residual racking price	2017$
*p*_*oe*_	Other el. hardware price	2017$
*c*_*PII*_	PII fees	2017$
*c*_*sc*_	Supply chain costs	2017$
*c*_*stax*_	Expenses for sales tax	2017$

**Table 2:** Variables in the cost equation for PV modules (see Eq 2).

Symbol	Meaning	Unit
*α*	Area utilization	unitless
*σ*	Solar constant	W_*dc*_/ m^2^
*A*	Wafer area	m^2^
$\eta_{m}$	Module efficiency	unitless
*y*	Yield	unitless
*v*	Silicon usage	m
*ρ*	Wafer density	g/cm^3^
*p*_*s*_	Polysilicon price	2017$/kg
*c*	Non-Si materials cost	2017$/m^2^
*K*	Plant size	modules/year
*K*_0_	Reference plant size	modules/year
*p*_0_	Capital, labor, O&M, and electricity costs per wafer for	2017$
	a plant of size K_0_	
*b*	Scaling factor	unitless
*p*_*mf*_	Module manufacturer margin	2017$/W

The margin accounts for the difference between module costs, represented by the first three cost components in Eq 2, and module factory gate prices. Note that in our analysis of innovations we only consider the variables and innovations affecting PV module costs (Eq 2), while the module factory margin is set by the factory owner and affected by market forces rather than by engineering advances or other technical innovations.

The level of detail represented in these cost equations is based on data availability. Cost equations could be written at more detailed levels capturing the specific innovations we examine qualitatively in this study if data were available at that level of detail. In that case, a single innovation would be represented as a change to a variable in a cost equation.

### 2.2 List of innovations and other factors affecting costs

In this work, we define an innovation as a successful commercial implementation of a new idea (including a first-of-a-kind idea as well as an existing idea that has been rearranged or repurposed) that improves technology performance [28–31]. Innovations represent significant changes in products or processes that often require deliberate efforts, such as research and development (R&D), to be developed and implemented [29, 32, 33]. In the cost modeling framework used here, where we refer to changes in the cost equation variables as ‘low-level mechanisms’ [12], innovations represent the detailed origins of changes to one or more variables. These innovations are studied qualitatively but could be examined quantitatively in the future in a more detailed cost model, where they would be represented as low-level mechanisms. Quantitative studies at this more detailed level are limited at present by data availability, though we outline approaches to estimating the cost effects of innovations in Section 3.6.

We select a set of innovations based on a process involving literature review and expert feedback. Our process is not meant to result in an exhaustive list of innovations, but rather is intended to result in a list of innovations that provide insight into the reasons for a technology’s cost decline. Arriving at a complete set of innovations is not possible until we decompose the technology into all of its inputs, down to the mined materials, and model their interactions in the entire economy. This framework serves as a starting point of an effort to identify a more comprehensive set of innovations.

For our PV example, we consider innovations affecting the costs of our two primary processes of interest: module manufacturing and PV systems installation. Since the inverter is a key component used in the installation process, we also consider several innovations affecting the manufacturing of inverters. In summary, we include innovations affecting the installation process, and innovations affecting the process of producing the two key components used in the installation process, the modules and the inverters. We do not include innovations affecting the process of producing the other components.

We first identify a set of innovations that improved PV’s performance since 1970 by conducting a literature review and through expert feedback (see S3 Appendix). If an innovation affected any cost equation variable, no matter which performance metric an innovation aimed to improve (e.g., decrease cost, increase reliability, improve consumer experience), it was included in the innovations table (S1 Table). After the literature review, the initial table of innovations was sent to experts in the PV industry to inquire whether the table comprehensively includes the key innovations. (Subsection 2.5 and S3 Appendix provide further detail on the expert feedback.)

We delineate innovations that affect PV module or system costs that range from more or less complex in that they may themselves depend on one or more interacting parts that together achieve cost improvement. For example, the development of the Czochralski method involved multiple distinct improvements (e.g., to the puller rod and the pulling method [34]). We summarize these step-by-step improvements as one innovation (‘Czochralski growth’) because they all served the same goal—growing single crystals by pulling a seed crystal out of molten silicon.

The number of innovations listed for each variable resulting from this approach is not meant to be an indicator of the degree of innovation or cost change that was achieved through that variable, since individual innovations do not necessarily affect costs equally and because of the inherent subjectivity in identifying key innovations. Instead, the innovations table is meant to provide a relatively comprehensive view of the innovations that are regarded as important in the literature and among experts. We note that this framework organizes innovations in such a way that they can be investigated in further depth in future work. Individual innovations can also be analyzed for their cost effects, as we demonstrate in Section 3.6.

In terms of geographical scope, our set of innovations is meant to represent a global overview for modules and inverters. Private and public institutions in many different countries contributed to innovations in PV equipment, and this equipment is traded globally. For innovations affecting installation processes (e.g., permitting), however, our focus is U.S.-centric, as these innovations often involve local and regional stakeholders and are not traded across countries in the same way PV equipment is. This choice was also motivated by the comparatively higher BOS costs in the U.S. markets [17]. Future work could explore innovations in lower cost markets.

In addition to specific innovations, we also identify other factors that cause changes in variables in the PV cost model. These factors include micro- and macroeconomic developments and their effects on commodity prices and wages, firm-level pricing decisions, factory-level learning and scale effects, as well as some changes in regulatory frameworks affecting the cost of PV. We call these factors ‘non-innovation drivers’ of cost change. (However, we note that some regulatory changes can be considered innovations, e.g., when they involve legal innovation as defined in Section 2.3.) One example of non-innovation drivers is learning-by-doing. When narrowly defined as improvements in performance due to repeating routine tasks, learning-by-doing does not require new ideas in order to affect costs. Another example is material price changes due to bulk purchases or market forces that affect the total material supply or demand.

### 2.3 Innovation typology

In this step, we develop an innovation typology to explain how an innovation has induced change in one or more cost variables. For PV innovations, we identify ten different innovation types. Process and component-level innovations have been studied in energy technologies [5] and in technologies more broadly [35], while studies of innovation through digitalization and automation have focused mostly on manufacturing, healthcare, and logistics industries (e.g., [36–39]). Here we draw on these studies and their respective definitions of innovation types, and we make adjustments to characterize the a wide set of cost-changing innovations in PV. For example, we distinguish between efforts to improve materials, component designs, and to simplify manufacturing and installation processes through component integration. The resulting typology includes broader (e.g., automation) and more narrow innovation types (e.g., component design change), which is tolerable in the context of our study because we do not derive conclusions from the number of innovations per type.

The definitions of the innovation types rely on the system boundaries reflected in Equation 1, which determine the processes studied in depth. In our PV example, our boundaries are drawn around both module manufacturing and PV system installation.

We assign one or more innovation types to each innovation. The assignment was performed by the research team based on the definitions provided below and then cross-checked within the team. An alternative approach would be to rely on an expert elicitation where experts categorize the innovations, or an automated system (e.g., based on text mining) can be developed to further standardize this process.

Rather than presenting a complete picture of the diverse innovation types contributing to PV’s cost decline, our classification should be interpreted as an initial proposal. Future work could identify additional innovation types through in-depth reviews of the history and contexts in which specific innovations emerged, and refine the typology by breaking down individual innovation types further.

We include the following innovation types for PV innovations:

- Material quality improvement: Development of new materials, use of new materials, or changes to existing materials that enable improved material performance, e.g., by reducing impurities and defects.
- Component design change: Functional changes of individual PV technology components.
- Component prefabrication and integration: Replacing on-site installation and manufacturing processes of individual PV components with previously designed and integrated components, often with the goal to enhance on-site or factory productivity and efficiency.
- Architectural change: Changes that affect the interaction of PV system components. These are often changes that focus on system performance (e.g., easier installation) in addition to component performance. Throughout the paper we define ‘architectural’ with respect to the architecture of the PV system as a whole and this type therefore applies exclusively to the BOS analysis, with only one exception where we relax our system boundaries and examine inverter manufacturing in greater depth and consider architectural changes to the inverter.
- Tool development: Development of new or improved hardware or software to complete a specific step in the processes required during module manufacturing, PV system design, or permitting.
- Process development: Application of new manufacturing or deployment (e.g., system design, installation, permitting) methods.
- Automation: Use of technology or machinery in lieu of manual labor to control and monitor a manufacturing or installation process; often changes aimed at reducing the need for human assistance.
- Digitalization: Use of digital instead of analog technology for hardware design, planning, configuration, and for communication purposes between human actors and technology components.
- Standardization: Establishing a limited set of solutions that will be repeatedly used by a number of parties; includes codifying best practices as well as development of technical standards.
- Legal innovation: Recombination of different elements of a right (e.g., the situations it applies to, the required burden of proof), or reinterpretation of a right leading to a new law.

### 2.4 Innovation time stamps and industry origins

In this step, we explore where the innovations originated and which industries contributed to the evolution of PV technology. An analysis of time stamps provides a chronological account of innovations in a technology’s evolution. We focus on the origins of PV innovations in this work; however, performing this analysis for several different technologies can reveal which technologies are better positioned to benefit from advancements in a broader range of fields.

Our approach to assigning innovations time stamps and industry origins follows five steps. (1) We select a set of keywords and phrases to describe each innovation in our list based on keywords appearing in papers and patents. (2) We conduct keyword searches on Google and Google Scholar to identify relevant sources. Within these sources, we search for direct statements on the industry that first commercialized or broadly adopted an innovation, as well as the time period. (3) For relevant papers, we conduct a backward citation search for information on the origins and time stamps. (4) We confirm our findings from steps (2)-(3) using the same keywords to search for relevant patents. (5) For relevant patents, we also conduct a backward citation search to rule out alternative industries of origin and time stamps. For innovations where we do not find relevant journal papers, we jump directly to steps (4) and (5) and use evidence from patents instead.

Using the above approach, external industries are assigned as the industry of origin if patents and publications on the PV invention (which led to the innovation) cite patents and publications on the invention with the same functionality used in another industry. Functionality refers to the use of similar architectures, components and/or mechanisms as in the innovation described in the innovations table. For instance, for high-frequency inverter designs, the functionality is the use of MOSFETs, IGBTs, and other devices to increase switching frequencies of inverters. We also assign an external industry if non-academic sources indicate that this industry used the innovation before PV (e.g., if a company outside the PV industry was the first to commercially implement an innovation). The innovation origins in our set are listed in Table 3. The subfields column in Table 3 shows the specific areas within the industry origins that we have found evidence of in our literature search. In line with previous work (e.g., [27, 40]) we conceptualize of PV innovations that originated in external industries and non-PV technologies as knowledge spillovers. We add to previously developed methods for studying inter-industry knowledge flows by describing spillovers based on the variables through which they act on technology costs. Variables represent features of equipment and manufacturing processes, and spillovers affect costs through innovations acting on equipment and processes shared across different industries, either at the same time or sequentially. While our dataset is insufficient to determine how important spillovers were for PV’s cost decline, it provides a starting point for answering this question. Future work could quantify the contribution of individual innovations, solicit expert feedback on the most important industries for individual innovations, and rank industries in terms of cost change impacts.

**Table 3:** List of industries and institutions (‘innovation origins’) assigned to PV innovations.

Origin	Subfields
Construction	Building design
	Structural stability testing
	Building materials
Electronics	Power electronics
	Microelectronics
	Energy wire manufacturing
Glass	Glass coatings
Semiconductors	Chips manufacturing
	Semiconductor device manufacturing
Metallurgy	Steel production
	Aluminum production
Photovoltaics	Module manufacturing
	System design
	Installation
	Equipment testing
Petroleum	Oil and gas drilling
	Oil and gas processing
Public institution	Energy commissions
	Professional associations

The data sources we use include academic literature (journal papers on specific inventions that led to innovations, review papers, history-focused sections in scientific papers, government reports including those submitted by research groups during reviews of their R&D grants) and gray literature (articles on company websites, market research reports, news media). Wherever possible we find multiple sources corroborating the time stamps and industries of origin.

The time period of innovative activities studied here is defined by the goal of this work—to identify the time periods during which technical novelties were first commercialized , thereby being transformed from inventions into innovations. We therefore consider time periods that were significant for PV’s commercial development, rather than focusing on the early scientific origins of photovoltaic systems (such as the discovery of the photovoltaic effect in the 1800s).

### 2.5 Ethics statement

Expert feedback was collected through emails and phone interviews between July 2018 and October 2018. The protocol was considered exempt after review by the Committee On the Use of Humans as Experimental Subjects of Massachusetts Institute of Technology. Research qualifies for an exemption if it is no more than minimal risk and it fits into at least one of the exemption categories in the federal IRB regulations. Exempt research is not subject to formal informed consent requirements and does not require signed consent. In this exempt research, we gathered feedback from industry experts in a peer reviewer capacity, rather than as experimental subjects. The focus of the feedback was only on the products, methods, policies, procedures, organizations, and not about the persons involved. Nevertheless, we provided information on the research to give the experts the opportunity to choose whether or not to participate in the research: In our communications with the experts, we provided a general description of the study and our initial innovations table. We informed the experts that we would use their voluntary feedback to guide our research. We asked the experts whether our table covers the most important innovations, whether the relationships between the variables and innovations were identified correctly, and whether the industry origins and timelines of innovations were accurate. After providing this information, we received input from several experts who voluntarily contributed their feedback. We anonymized the feedback, i.e. expert names are not matched with any specific comments, in this paper.

## 3 Results

### 3.1 General observations

**Fig 1:**
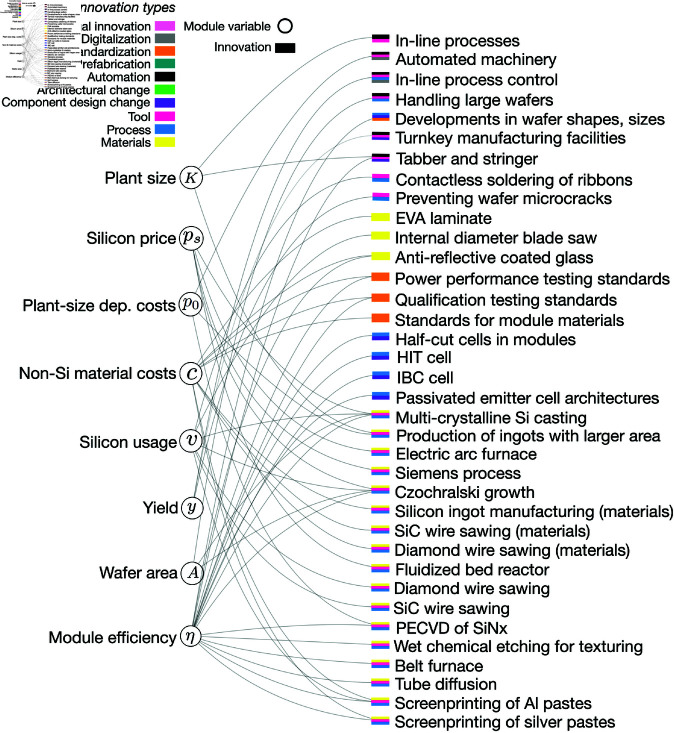
PV module innovations. Module variables (circles) and innovations (rectangles) affecting variables. Innovations (rectangles) are color-coded based on their types or type combinations, and shown in the order of most prevalent (top) to least prevalent (bottom) type or type combination. Abbreviations: Al = aluminum; PECVD = plasma-enhanced chemical vapor deposition; SiNx = silicon nitride; SiC = silicon carbide; IBC cell = interdigitated back contact cell; HIT cell = heterojunction with intrinsic thin-layer cell; EVA = ethylene vinyl acetate.

**Fig 2:**
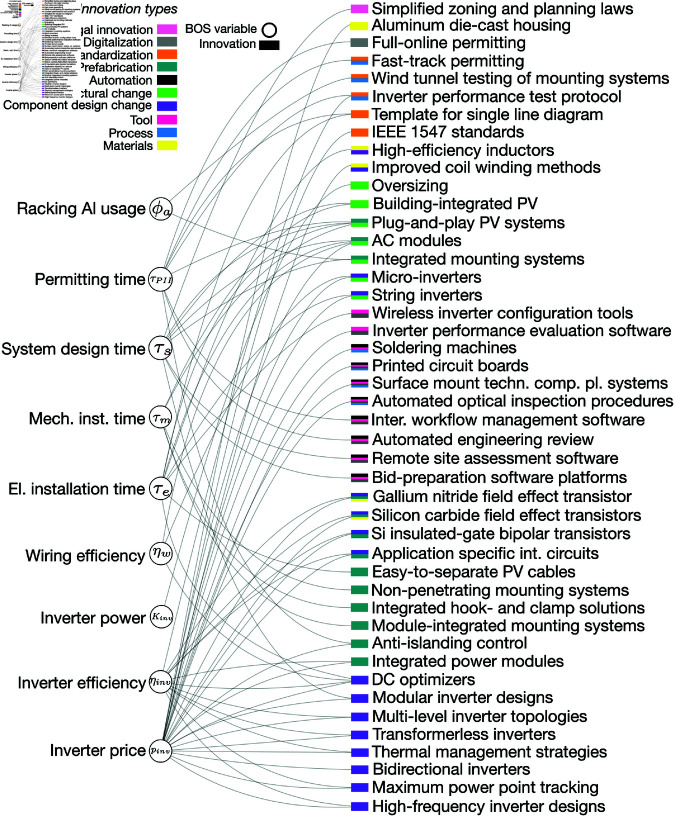
BOS innovations. BOS variables (circles) and innovations (rectangles) affecting variables. Innovations (rectangles) are color-coded based on their types or type combinations, and shown in the order of most prevalent (top) to least prevalent (bottom) type or type combination. Abbreviations: DC = direct current; int. circuits = integrated circuits; comp. pl. systems = component placement systems; AC = alternating current.

One of the main outcomes of applying this framework is a list of innovations affecting the cost of PV systems that demonstrates the diversity of efforts that contributed to this technology’s development. Many different fields of scientific research and areas of engineering and practice are represented. Our innovations table lists 81 unique innovations that affected PV system costs since 1970 (see Figs 1 and 2 and the innovations table shown in S1 Table). Note that the table has 130 entries. This includes 81 innovations, some of which affect multiple variables and therefore are listed under multiple variables, and 49 non-innovation factors. Roughly half of these innovations influenced module-related variables, while the other half affected BOS-related variables. Using alternative definitions of specific innovations may alter the number of items in our list and the distribution of innovations across module and BOS-related variables. We therefore use the itemized innovations table predominantly as a starting point for qualitative observations. Our main conclusions are not derived from innovations counts.

We identify multiple innovations for all variables in the PV system cost equation. In particular, the variables causing a larger fraction of PV’s cost decline, module efficiency and inverter price [11, 12], are linked to a larger number of innovations (Figs 1 and 2). Although the number of innovations identified for a variable is not necessarily correlated with the variable’s cost change impact, the multiplicity of innovations identified for these variables potentially indicates the extent and efficacy of R&D efforts devoted to these important variables. In addition, some of these innovations such as Czochralski growth are among the more broad innovations in the table, which include several smaller scale innovations, e.g., the use of seed crystals to define crystal orientation in Czochralski growth, liquid encapsulation techniques, or optical sensing to control crystal diameters [41]. This suggests that these variables were targeted by several simultaneous improvements that were coordinated and integrated to achieve higher performance. Many innovations in our table influenced two or more variables, reflecting the coupling of variables through manufacturing processes or component designs (e.g., inverter designs). We observe such innovations mainly for the variables inverter price and inverter efficiency, due to improvements that increased power density and thereby reduced materials needs for heat sinks and associated costs. Wafer thickness and silicon utilization also share a number of innovations (e.g., silicon carbide and diamond wire sawing).

### 3.2 Innovation types

Characterizing innovations helps us to better understand the sources of technological change. We learn valuable lessons on the innovation types affecting different technologies, different parts of the same technology, such as modules and BOS in our PV example, and different cost variables. Below, by discussing specific examples from our PV innovation typology, we illustrate some of the important insights one can gain from this qualitative analysis.

The innovation types observed differ for modules and BOS, as indicated by the color coding in Fig 1, which shows innovations affecting module costs by innovation type, and Fig 2, which shows innovations affecting BOS costs. As shown in Fig 1, module innovations mainly led to new developments in materials, tools and processes. For example, growing higher quality single crystal silicon wafers without defects enables better absorption of light and increased conversion efficiency. Tools such as wire saws enabled cutting silicon ingots into thinner wafers, reduced material losses in the process, and greatly increased the speed of the wafer slicing process compared to the previous technology, internal diameter sawing. As an example of process development advances, screenprinting thin strips of metal on the silicon wafer reduced equipment costs and increased throughput.

For BOS, many innovations were classified as ‘component design change’ and ‘prefabrication’ (Fig 2). Examples of component design changes include transformerless inverters, modular inverters, and string inverters. These innovations aim at improvements such as reducing material use (transformerless inverters), reducing the need for customized manufacturing steps for different inverter applications and designs (modular inverter designs), and limiting electrical losses (string inverters). Examples of component prefabrication include application specific integrated circuits or AC modules. Separate inverter or module mounting system components were combined into integrated pieces of equipment, making manufacturing and installation simpler.

Despite the diversity of innovation types affecting module and BOS variables, we see some patterns in how innovation types combine. To improve materials, module-related innovations often involved the development of new processes (color-coded yellow-blue in Fig 1), or the combined development of new processes and tools (color-coded yellow-blue-pink in Fig 1). Examples for material-process-tool innovations include wet chemical etching for texturing, the use of belt furnaces, tube diffusion, and the development and screenprinting of silver and aluminum pastes. Innovations that increased automation in module manufacturing also involved the simultaneous development of new processes and tools (color-coded green-blue-pink in Fig 1. Combinations of innovation types are more diverse for BOS than for modules, with combinations of component design change and prefabrication, component design change and architectural change, and automation and digitization appearing several times.

Also noteworthy are the least prevalent innovation types: architectural changes, digitalization, standardization, and legal innovation. The scarcity of innovations falling into these categories may indicate a potential for improvement in these areas. Another interpretation is that these innovation types require non-traditional settings; standardization and architectural changes are often developed with the cooperation of different institutions and component providers, rather than by individual companies in isolation. A third possible interpretation is that the least prevalent innovation types are those that are less likely to lead to cost reductions. For example, legal innovations may target the increase of biodiversity on solar sites but may not lead to cost reductions (without accounting for externalities).

**Fig 3:**
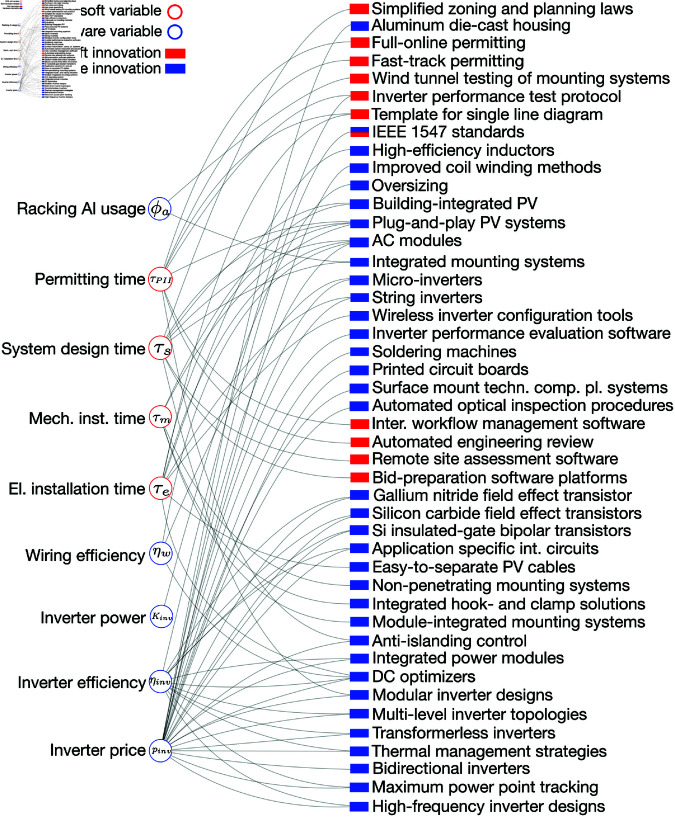
Hardware and soft BOS innovations. BOS variables (circles) and innovations (rectangles) affecting variables. Variables and innovations are color-coded based on whether they represent physical (‘hardware’, shown in blue) or non-physical (‘soft’, shown in red) technology characteristics and changes therein. In accordance with our system boundary we do not consider soft technologies in module manufacturing. All module variables are classified as ‘hardware’ and are not shown here. Note that most innovations fall into one of the two categories ‘hardware’ and ‘soft’. However, we classify the innovation ‘IEEE 1547 standards’ as related to both hardware and soft technology because it led to changes in PV deployment processes (e.g., interconnection testing) and in PV hardware (e.g., inverter functionalities).

Finally, we parsed the table in terms of innovations affecting physical or ‘hardware’ variables, and innovations targeting non-physical or ‘soft’ variables (see Fig 3). We term the variables that characterize physical components such as module efficiency, area, and part counts ‘hardware variables’, while the variables that do not characterize physical components are termed ‘soft variables’, and include variables such as task durations, wages, and fees [11].

The vast majority of PV innovations in our table are hardware innovations. ‘Soft’ innovations are less prevalent and target soft technology, which we define as practices that define processes [11]. Several of these were developed during the past decade, such as fast-track permitting processes, standardized templates for single-line diagrams, and automated engineering reviews of grid interconnection applications. It is also noteworthy that most innovations target either hardware or soft variables, though a few examples affect both (e.g., railless mounting systems).

Note that the hardware vs soft technology variable distinction was applied only to BOS cost variables in our model and not to module cost variables. This is due to the observation that soft costs are an impediment to BOS cost declines, whereas they appear not to have impeded the steady cost decline in module costs [11, 18, 42]. Applying the distinction to BOS enables a discussion of a diverse set of components and deployment procedures that affect BOS costs.

### 3.3 Innovation origins

Analyzing the industry origins of each specific innovation allows us to develop a better understanding of the knowledge spillovers benefiting PV system costs. The variables in our cost equation represent the cost of materials and processes developed, used, and improved by a variety of industries; through these variables and the corresponding innovations we develop a bottom-up picture of PV’s position in a broader ecosystem of technology industries.

Looking across modules and BOS, the semiconductor and electronics industries affected many innovations we considered, suggesting that these industries had a significant impact on PV systems. Several important PV module innovations originated in the semiconductor industry (e.g., Czochralski growth, PECVD, belt furnace, tube diffusion, contactless soldering of ribbons). We also identified innovations with roots in metallurgy, electronics, and petroleum industries as well as the PV industry. Looking at BOS, inverter prices benefited from inventions in the semiconductor and electronics industries. Other BOS variables were influenced by innovations with roots in industries such as software engineering, electric utilities, PV, and electronics. Both module and BOS innovations origins show that PV was well-positioned within an ecosystem of technologies in many industries.

Several innovations were designated as originating in the PV industry in the sense that the technical know-how was originally developed by the PV industry. These innovations include half-cut cells in modules, heterojunction with intrinsic thin layer cells, string inverters, maximum power point tracking (MPPT), AC modules, and remote site assessment software to support system design. Also, for the majority of the innovations, the PV industry made contributions in adapting processes and tools from other industries. For example, using wind tunnel testing for the design of PV mounting structures required an adjustment of testing procedures to estimate and interpret aerodynamic loads for PV panels, which were not well described by the shapes tabulated in previously existing standards [43]. R&D efforts were therefore needed to determine how the results of individual tests of a given array on a particular roof structure could be generalized to derive design guidelines for an entire class of roof-mounted solar arrays. As another example, the process of screenprinting for metallization was based on the technology borrowed from the thick-film electronic circuitry industry [44–46]. New silver pastes were developed for PV since most pastes available in the 1970’s and 80’s for thick-film technology were not suitable for a silicon surface [47, 48]. Developing new pastes that can minimize contact resistance and incorporate alternative materials required dedicated research efforts by the PV industry to understand the contact forming process [49]. Anti-reflective coatings for glass provide yet another example of an external innovation that the PV industry developed further for its own purposes. Although extensive research has been done outside the PV domain to reduce the reflectivity of glasses used in optical equipment, ensuring the durability of coatings under outdoor weather conditions faced by PV panels required the development of additional coating characteristics, as well as performance testing to document energy gains and soiling behaviors of new coatings [50, 51].

Overall, PV industry-based innovations were about equally prevalent for modules and BOS. This might indicate that both component-level and system-level innovations required inputs from the PV industry as well as other industries. In terms of the actors and institutions involved in the invention and innovation process, we see a larger diversity for BOS as compared to modules. While most module innovations originated in research organizations or in industry, several BOS innovations were developed by city governments, states, or PV industry associations (e.g., State of Colorado, IEEE, U.S. municipalities).

### 3.4 Timeline of innovations

**Fig 4:**
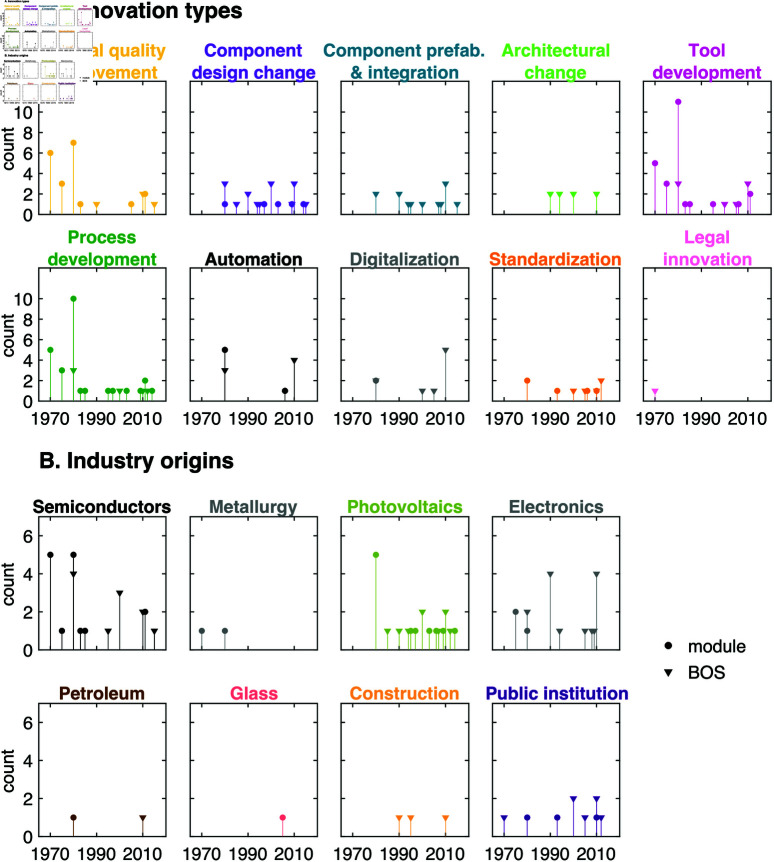
Innovation types and industry origins. The number of innovations of each innovation type (panel A) and industry origin (panel B) are plotted over time based on approximate innovation years. Note that an innovation might be assigned to more than one innovation type, and therefore might be counted under multiple innovation types. Module innovations are shown with circles, while BOS innovations are shown with triangles. The colors of the innovation types in panel A match the colors in Fig 1. The module analysis considers a global scope while the BOS analysis is focused on the US (see methods). Note that some innovations may be larger in scope than others, such that changing the scope of an innovation could affect annual innovation counts.

Our analysis suggests that innovations were spread throughout the period since the 1970s (Fig 4). For module innovations, the ’70s and ’80s seem to have been particularly productive in this sense. Several module-related inventions were commercially implemented in the PV industry during that time period (e.g., wire sawing, development and screenprinting of aluminum and silver pastes).

Besides providing a chronological account of the innovations, when this analysis is combined with the previous analyses described earlier, it helps uncover the trends in innovation types and industry origins over time. Fig 4 indicates that some innovation types are concentrated in certain time periods. For example, architectural change, digitalization, and component prefabrication and integration appear mostly after the 1990s. Module-related innovations are more prevalent in the earlier periods, and are concentrated in a group of industries (i.e., PV, metallurgy, semiconductors, electronics). On the other hand, BOS innovations appear more frequently in the later periods and are spread across the industries except for mining, metallurgy, and glass. The module analysis considers a global scope while the BOS analysis is focused on the US (see methods 2.2).

### 3.5 Non-innovation drivers

Non-innovation drivers that changed the cost variables show some differences across modules and BOS. For modules, non-innovation drivers have been mainly related to cheaper materials and equipment (e.g., material discounts due to bulk purchases, easy access to materials and equipment due to a maturing PV industry) as well as learning-by-doing. For BOS, there was less evidence of these bulk purchasing discounts and easy access to supplies, though we acknowledge that these effects likely exist to some extent. For BOS, drivers such as regulations were important, but often changed in a deliberate way, with the intention of improving PV costs, and thus we consider them to be innovations.

We also identified non-innovation drivers common to both modules and BOS. One such driver that was assigned to several variables for both modules and BOS was learning-by-doing. This assignment relies mainly on the assumption that repetition leads to learning and improvement, but it is difficult to prove this effect with data. Although there may be innovations that enable factory-level or installer-level learning-by-doing, we did not find evidence for such innovations. Other non-innovation drivers that are common to BOS and modules come from changes determined outside the PV industry, such as wages and material price changes due to demand from other markets.

### 3.6 Estimating the magnitude of cost change due to specific innovations

Several approaches can be taken to move beyond a qualitative assessment and toward a quantitative assessment of the cost impact of a specific innovation. The three approaches we describe here can be used to rank PV-related innovations according to their effect on costs, though achieving this would require extensive further research. Quantifying the cost impacts of a ‘complete’ set of innovations is not possible until we dissect the technology into its most basic inputs and track the innovations affecting the cost of these inputs throughout history. This is likely impossible given data limitations and funding constraints in real-world research settings, but the approaches discussed here may support further progress by enabling more targeted model development and data collection.

One approach to estimating the cost impacts of an innovation is to track changes to variables in the cost equation directly before and after an innovation occurred, to assign cost change to a particular innovation’s effect on one or more cost variables. This approach can be taken if no more than one innovation significantly affected each variable in the cost equation.

A second approach would be to model the effects on cost equation variables of multiple innovations occurring simultaneously. For this, a physics-based model can be used to further decompose the effects of innovations on specific variables. This can be challenging to do in a retrospective assessment, however, since it may be difficult to collect data on the exact timeline and nature of all innovations occurring simultaneously. However, this can more easily be done in prospective assessments, using physics-based models that capture sets of possible combinations of different innovations [52, 53].

A third approach is to go part of the way toward a model of how simultaneous innovations affect a particular cost variable by breaking cost variables into additive parts and assigning approximate weights to the innovations affecting these parts, either retrospectively, as is the focus of this paper, or prospectively. In this section we demonstrate approaches one and three. (In other forthcoming work, we demonstrate approach two for the case of lithium-ion battery technologies [53].)

#### Example 1: Estimating the cost impact of an individual innovation.

In this example, we demonstrate approach one and estimate the cost impact of a particular innovation, namely wire sawing. Through this example we see how a single innovation can have a significant impact on cost. Since wire sawing was introduced in the 1980s [54, 55], we model its cost impacts based on the state of the technology around that time. First, we determine based on a literature review that silicon usage (*v*) and throughput, i.e. plant size (*K*) which we measure as number of modules per year, were the main variables that were impacted by the switch to wire sawing from the pre-existing technology, the internal diameter saw [56, 57].

To obtain the cost effects of this switch, we first populate our module cost equation with data for the state of the manufacturing before the switch, circa 1980 (see S2 Table in the appendix). We then change the silicon utilization and plant size variables by the amounts indicated by the literature [55–57]. Finally, we estimate the impact of these changes on module cost by using the cost change method developed in [12].

**Fig 5:**
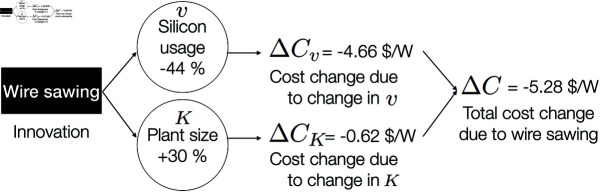
Example 1: The effect of wire sawing on cost variables and the resulting cost change around 1980 (2015 USD). Overall, these changes lead to a cost decrease from 29 $/W in 1980 to 24 $/W (2015 USD). A 44% reduction in silicon usage contributed 88% of this cost change, while a 30% increase in plant size (measured in the number of modules produced per year) contributed 12%. The cost change due a change in a variable is calculated by using the cost change method developed in [12].

Fig 5 illustrates the approach and shows the effect of wire sawing on cost variables and the resulting cost change. Reducing the silicon losses during wafering by 30%, wire sawing reduces the module cost by 4.66 $/W (2015 USD). By increasing the throughput (i.e. plant size measured in number of modules per year) by 30%, it reduces the module cost by an additional 0.62 $/W (2015 USD). Overall, these changes lead to a cost decrease from 29 $/W in 1980 to 24 $/W (2015 USD). Silicon usage reduction contributed 88% of this cost change, while increased throughput contributed 12%. (Please see S1 Appendix for details on the data and methods used in this example.)

In this example, we focused on two variables, silicon utilization and plant size that improved due to wire sawing. However, an innovation may initially change certain variables such as yield in a direction that increases cost.

For example, cutting thinner wafers reduced throughput in earlier versions of wire saws due to reduced silicon ingot load and cutting speeds [58]. Thinner wafers were also harder to handle in subsequent processing steps including demounting, signaling, and cleaning [58], and therefore reduced yield [59]. Also, the cost of consumables used in wire sawing (e.g., wires, slurry, glue) was still higher than that of the ID saw until the mid-1990s [60]. To achieve higher yields and higher wafer quality, the wire sawing process was optimized by controlling the wire speed, wire tension, cutting speed, composition and properties of the abrasive SiC powders and the carrier fluids, and various other machine parameters [61]. This indicates that the cost effect of an innovation likely evolves as the processes are optimized over time, as exemplified by this historical account of wire sawing. The approach presented here can be used to capture the intermediate or final cost impacts of an innovation.

#### Example 2: Attributing total cost change to innovations.

This section demonstrates approach 3, an approach to quantitatively estimating the cost reductions resulting from multiple innovations affecting a given cost variable. In this approach, we break a cost variable into additive parts and assign approximate weights to the innovations affecting these parts. In this demonstration, we will focus on the innovations affecting a module-related variable, non-silicon materials costs, *c*, in the module cost equation (Eq 2).

**Fig 6:**
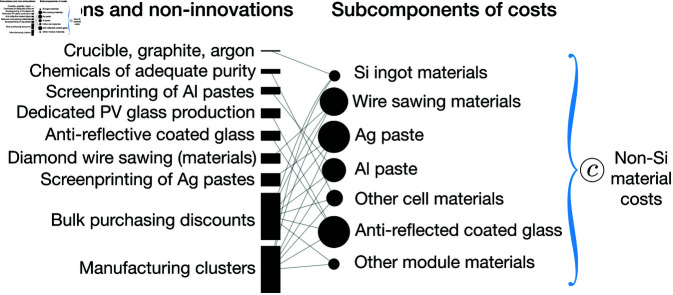
Example 2: Demonstration of a method for further, more detailed analysis of cost change drivers. Cost reduction due to a change in a cost variable, *c* (non-silicon materials costs), is broken down into contributions of different subcomponent material categories between 2010 and 2018 (shown on the right). The subcomponent materials are matched with the innovations affecting them (developments in ingot materials such as crucible, graphite, argon, diamond wire sawing, screenprinting of Ag and Al pastes anti-reflective coated glass) as well as a range of non-innovation drivers (easier access to chemicals of adequate purity, manufacturing clusters, bulk purchasing discounts). Rough estimates of the cost changes are then made by assuming that within each subcomponent all innovations and non-innovation drivers contribute equal proportions to the total cost reduction observed for each subcomponent of the non-silicon material costs variable. In this figure, cost change due to each subcomponent material, innovation, or non-innovation is proportional to the area of the shape that appears next to it.

We first disaggregate the non-silicon materials costs variable into its additive subcomponents in order to obtain a fine-grained picture of the specific innovations affecting them. The subcomponents include the costs of materials needed to produce silicon ingots, including crucibles and argon, SiC or diamond wire sawing materials, silver and aluminum pastes and associated screenprinting materials, anti-reflective coated glass, and other cell and module materials. The next step is to assign cost reductions to the innovations and non-innovation drivers associated with variable *c*. We first obtain the cost reduction in the subcomponent materials between 2010 and 2018 using data from the literature [42, 62, 63] and our cost model for PV modules [12], as described in detail in the S2 Appendix. We then take the innovations and non-innovation drivers affecting variable *c* from our innovations table (S1 Appendix) and match them with the subcomponents of *c* as shown in Fig 6. Note that some of the innovations that we listed in the innovations table which affect non-Si materials costs such as EVA laminate, developments in SiC wire sawing, qualification testing standards, and standards for module materials are not included in this analysis because they have minimal effect on cost during the time frame studied in this example (2010-2018). To demonstrate the method, here we apply the rough estimate that the cost reduction caused by each subcomponent material category is equally distributed across the innovations affecting that category.

Based on these rough estimates, the largest overall contributors to cost reductions in the 2010-2018 period were bulk purchasing discounts and manufacturing clusters, both of which are non-innovation drivers (see left column in Fig 6, where cost change is proportional to the area of each rectangle). In this example, we assumed that these non-innovation drivers affected many or all subcomponent material categories. Due to this assumption and our equal distribution of cost effects across innovations or non-innovations, they resulted in a large cost impact.

In future work, cost impacts could be distributed in a non-equal way depending on data availability. Note that the example shown here is intended as a demonstration rather than a definitive quantitative assessment of cost impacts. In this example, we estimated the effects of innovations and non-innovations through changing the non-silicon materials costs (*c*) variable only. Some of these innovations also affect other variables and thus lead to other cost changes as well. These other variables also would need to be taken into account in order to estimate the total cost impacts of an innovation.

## 4 Discussion

This paper presents a framework for identifying and characterizing specific innovations that affect the cost of a technology. We present an approach to qualitatively linking innovations to cost change and quantitatively estimating their effects where appropriate data exists. In this way our framework helps develop further understanding of the nature of cost-reducing technological innovation. Here we examine PV, a major carbon-free technology that declined rapidly in cost, but the method can also be applied to other technologies. A cost equation serves as a guide to highlight innovations with direct effects on cost-related variables. The equation provides a structure for sorting through and analyzing a rich history of diverse efforts to improve PV. Moreover, applying the framework to PV sheds light on the limitations faced in quantifying innovation drivers. Some of these limitations can be addressed through further data collection and modeling, as we discuss.

For PV, we find that a diversity of innovations affected both PV modules and BOS, with certain revealing differences in innovation patterns and stakeholders involved. Technological progress in PV modules was largely a result of new, highly specialized manufacturing processes, with clear patterns of innovation that combine new tools and processes to improve materials quality or automate manufacturing steps. In contrast, innovation patterns in BOS were more difficult to find, aside from a concentration on electronics innovations affecting the design of inverter components and a more recently emerging emphasis on ‘soft’ innovations, such as efforts toward fast-track permitting post-2010. Our results also shed light on knowledge spillovers between PV and other technologies, with many PV innovations originating in the semiconductor and electronics industries.

Similarities between our results and previous compilations of PV innovations indicate that our table covers many module innovations considered influential based on patent citation analysis (e.g., [5, 64]), while also including a larger and more diverse set of BOS innovations. However, the list of innovations presented in this study is not meant to be exhaustive. Rather, it is intended as a starting point for preparing and investigating an extensive dataset of innovations affecting each cost variable.

A limitation of this study is the small number of experts consulted to validate our innovation dataset. Future work could use the approach outlined here to compile an initial list of innovations and subsequently invite a larger set of experts to critique the list in an iterative manner, progressing from version to version until no more innovations are added or removed. This approach could gradually reduce bias in the resulting sample of innovations, as any human expert, by definition, focuses on a certain area and can only evaluate the comprehensiveness of an innovation list with respect to that area. The framework presented here can also be used to structure high-quality expert elicitations [65] that probe the knowledge of many different experts, from engineering and other fields, at a level that directly matches their expertise.

Importantly, this study provides a new perspective on the nature of knowledge spillovers across technologies. The degree to which a technology or product is similar to and can benefit from other technologies is dependent on the physical processes they share in common. Modeling the characteristics of these processes as cost variables, and using variables as a starting point to identify innovations and industry origins, helps shed light on spillovers from a physics and engineering perspective not previously applied in the literature on knowledge spillovers. Physics-based approaches can also be employed to develop strategies for leveraging spillovers based on the processes a technology shares with others [66]. Studies like this one could be used to help identify networks of technologies and industries relying on similar physical processes to understand in advance whether some technologies are better or worse positioned to benefit from spillover . In the case of PV, the technology (both modules and BOS) was well-positioned within an ecosystem of continuously advancing technologies in many industries (semiconductors, mining, electronics), which may have contributed to PV’s rapid cost decline. Identifying this position in advance may help with technology forecasting and priority setting for R&D investments.

These findings add to existing hypotheses on the reasons for PV’s steep cost trajectory. PV modules not only benefited from many improvements to cost-determining variables from within the PV industry [11, 12], but were also influenced by a diverse set of industries outside of PV.

Overall, our findings are in line with existing knowledge of the nature of innovation in energy technologies, which involve chemical, mechanical and electrical processes, and therefore inherently draw on inventions from multiple technological domains in order to evolve in performance [27]. However, taking a look at the diverse set of specific innovations that were important shows just how significant this was.

Consistent with conclusions that were drawn in earlier work, many spillover innovations required adaptation to be applicable to PV, and thus the policy support for PV was critical in driving the innovative activity that led to PV’s exceptionally rapid and sustained cost decline [12]. Market-expansion policies may have been especially important in experimenting with adapting and applying innovations from other industries to the PV module manufacturing and installation processes.

The specific innovations identified and characterized using this framework provide a useful starting point for investigating a range of higher-level mechanisms that influence technology costs. These higher-level mechanisms include human efforts such as R&D and others mechanisms not as directly linked to innovation such as learning-by-doing, and economies of scale. Delving into detailed, specific innovations provides a way to understand the nature of these higher-level mechanisms and attribute cost change to them. We can learn about the different job descriptions that were involved, and sometimes even infer the specific locations, companies, and individuals that contributed, where data is available. The importance of public policy in driving these higher-level mechanisms can then also be studied.

The method we present in this paper can also be adapted to arrive at prospective models of innovation and cost change. To do so, the interactions between components of a technology must be modeled to determine whether any given innovation affecting multiple technology components will allow for the technology to function and will bring about a desirable change overall. Considering the interactions between individual innovations, where several are under consideration, is also important. Using a design structure matrix and engineering knowledge is one approach to this sort of prospective assessment [67, 68].

Another direction for future research is to expand the study of the life-cycle of innovations from their origin to their evolution beyond the PV industry. While we discuss and document the contributions from external industries to PV’s cost evolution, innovations adapted by the PV industry may in turn have contributed to further innovations outside the PV industry.

## Supporting information

S1 Text Nature of innovations affecting photovoltaic system costs.
(PDF)
