# Supplementary material for: Nature of innovations affecting photovoltaic system costs
Source: PLoS One. 2025 Aug 11;20(8):e0320676. doi: 10.1371/journal.pone.0320676 (PMC12338801; doi:10.1371/journal.pone.0320676)
Supplement: S1 Text — Nature of innovations affecting photovoltaic system costs. (PDF) [file pone.0320676.s001.pdf]

Supporting Information: Nature of innovations affecting photovoltaic system costs

Goksin Kavlak[1]◑, Magdalena M. Klemun[1,2]◑, Ajinkya S. Kamat[1], Brittany L. Smith[3], Robert M. Margolis[3], Jessika E. Trancik[1,4]*

1 Institute for Data, Systems and Society, Massachusetts Institute of Technology, Cambridge, MA, USA
2 Department of Civil and Systems Engineering, Johns Hopkins University, Baltimore, MD, USA
3 National Renewable Energy Laboratory, Washington, DC, USA
4 Santa Fe Institute, Santa Fe, NM, USA

◑These authors contributed equally to this work.
* trancik@mit.edu

S1 Table. Innovations table. S1 Table (next page) contains the set of innovations and non-innovation drivers identified in this work.

Number	Variable	Variable_ moduleOr BOS	Innovation_name	Innovation_description	Innovation_type	Industry	Time_innovation
**1**	**eta**	**module**	Czochralski growth for mono-crystalline Si	Produces Si ingots. Improvements: Better control of crystal growth process through improved hot zone design, magnetic fields, recharging, computer simulations, preventing crucible contamination leading to higher quality crystals, larger ingots, and reduced material losses from cropping and shaping the ingot.	Material quality improvement; Process development; Tool development	Semiconductors	1970
**2**	**eta**	**module**	Multi-crystalline Si casting	Produces Si ingots. Improvements: Crucible coatings, seeding of silicon to obtain optimum grain sizes, gettering leading to higher quality and larger ingots, and reduced material losses from cropping and shaping the ingot.	Material quality improvement; Process development; Tool development	Metallurgy	1980
**3**	**eta**	**module**	Wet-chemical etching for texturing	Improving optical performance, and reducing wire saw damage. Improvements: Etch solutions with reduced impurities, in-line etch process, limiting surface damage	Material quality improvement; Process development; Tool development	Semiconductors	1975
**4**	**eta**	**module**	Belt furnace	Junction formation process. Improvements: Spin-on, CVD, spray-on, screenprint deposition methods, process optimization (e.g. to match tube diffusion efficiency, for newer cell structures like PERC,…), automation, laser doping instead of IR lamps	Material quality improvement; Process development; Tool development	Semiconductors	1980
**5**	**eta**	**module**	Tube diffusion	Junction formation process. Improvements: P2O5 to liquid POCl3, Larger furnaces, longer tubes, 2-step instead of 1-step process, low-pressure version, process optimization (e.g. of oxygen flow, for shallow junction, with new Ag pastes, for newer cell structures…), automation. Many versions of process are in use.	Material quality improvement; Process development; Tool development	Semiconductors	1970
**6**	**eta**	**module**	PECVD of SiNx for surface passivation	Depositing layer(s) for surface passivation and antireflective coating. PECVD of SiNx is a key innovation due to simplification of process by replacing multiple steps for passivation, AR coating, and paste control. Rear surface passivation is now emerging in PERC.	Material quality improvement; Process development; Tool development	Semiconductors	1983
**7**	**eta**	**module**	Development and use of screenprinting of silver pastes	Silver paste forms metal contacts; allowed less doping with less capital intensive (screenprinting) equipment and increased throughput. Development of silver paste compositions and development of screenprinting process were key innovations.	Material quality improvement; Process development; Tool development	Electronics	1975
**8**	**eta**	**module**	Development and use of screenprinting of aluminum pastes	Aluminum paste forms metal contacts at the back of the cell and creates back surface field (Al-BSF) architecture. Development of paste compositions and development of screenprinting process, especially the Al-BSF formation, were key innovations.	Material quality improvement; Process development; Tool development	Electronics	1975
**9**	**eta**	**module**	Passivated emitter cell architectures: Passivated emitter solar cell (PESC), Passivated Emitter Rear Locally diffused cell (PERL), and Passivated emitter and rear cell (PERC)	Surface passivation to reduce recombination; higher rear surface reflection	Component design change; Process development	Photovoltaics	2009
**10**	**eta**	**module**	Interdigitated back contact cell (IBC)	Reduced shading losses, simpler interconnection, higher packing density, lower resistive losses, but several costly high temperature processes	Component design change; Process development	Photovoltaics	2003
**11**	**eta**	**module**	Heterojunction with intrinsic thin layer cell (HIT)	a-Si:H and c-Si create the pn-junction; higher efficiency at high temperatures, low process temperatures	Component design change; Process development	Photovoltaics	1997
**12**	**eta**	**module**	Tabber and stringer	Connecting solar cells. Key innovations include: Automation of stringing which reduced operation time and energy; machine vision for optical alignment of cells allowing narrower and more busbars reducing interconnect resistance, and detecting defects.	Automation; Tool development	Photovoltaics	1980

**13**	**eta**	**module**	Anti-reflective coated glass	Helps with light trapping improving power output. Innovation for PV was AR coating with reliability and durability needed for outdoor PV	Material quality improvement	Glass	2005
**14**	**eta**	**module**	Developments in wafer shapes and sizes	Quasi-square or rectangular shaped wafers and reduction in wafer size variability allowed the gap between cells to be reduced, increasing the packing factor of the module. Enabled by improvements in crystal growth and other wafer processes.	Component design change; Process development; standardization	Photovoltaics	1980
**15**	**eta**	**module**	Half-cut cells in modules	Reducing resistive losses; higher shading tolerance	Component design change; Process development	Photovoltaics	2014
**16**	**eta**	**module**	Qualification testing standards	IEC 61215 standards outline test procedures for design qualification mainly aiming for production quality control. Enabled balancing rigid quality requirements and allowed the development of low-cost modules. IEC 61730 outline PV module safety standards consistent with IEC 61215.	Standardization	Public institution	1993
**17**	**eta**	**module**	LBD	Improvements in wafer, cell, module manufacturing by repetition of routine tasks	Non-innovation		
**18**	**t_Si**	**module**	Internal diameter blade saw	Cutting ingots into wafers. Allowed flat and parallel cuts with little wafer breakage.	Material quality improvement	Semiconductors	1970
**19**	**t_Si**	**module**	SiC wire sawing	Cutting ingots into wafers. Wire diameter, type, and cutting speed determine the material usage. Improvements: Increased speed compared to internal diameter blade saw, thinner and stronger wires over time, reduced kerfloss	Material quality improvement; Process development; Tool development	Semiconductors	1980
**20**	**t_Si**	**module**	Diamond wire sawing	Cutting ingots into wafers using wires coated with diamond particles. Mainly used for monocrystalline wafers as of yet. Improvements: Ability to reduce wafer thickness and further reduce kerfloss.	Material quality improvement; Process development; Tool development	Semiconductors	2011
**21**	**t_Si**	**module**	Contactless soldering of ribbons	Prevents microcracks by creating homogeneous temperature field within the cell, reducing thermal and mechanical stress; helped overcome limitations in handling and processing thinner wafers. Types include IR soldering, laser soldering.	Process development; Tool development	Semiconductors	1985
**8**	**t_Si**	**module**	Development and use of screenprinting of aluminum pastes	Aluminum paste forms metal contacts at the back of the cell and creates back surface field (Al-BSF) architecture. Development of paste compositions and development of screenprinting process, especially the Al-BSF formation, were key innovations. New Al paste formulation that reduce wafer bow (warpage) after firing enabled thinner cells.	Material quality improvement; Process development; Tool development	Electronics	1975
**22**	**t_Si**	**module**	LBD	Improvements in wafer, cell, module manufacturing by repetition of routine tasks	Non-innovation		
**18**	**U_Si**	**module**	Internal diameter blade saw	Cutting ingots into wafers. Allowed flat and parallel cuts with little wafer breakage.	Material quality improvement	Semiconductors	1970
**19**	**U_Si**	**module**	SiC wire sawing	Cutting ingots into wafers using wires coated with diamond particles. Wire diameter, type, and cutting speed determine the material usage. Improvements: Increased speed compared to internal diameter blade saw, thinner and stronger wires over time, reduced kerfloss	Material quality improvement; Process development; Tool development	Semiconductors	1980
**20**	**U_Si**	**module**	Diamond wire sawing	Cutting ingots into wafers, mainly used for monocrystalline wafers as of yet. Improvements: Ability to reduce wafer thickness and further reduce kerfloss.	Material quality improvement; Process development; Tool development	Semiconductors	2011

**1**	**U_Si**	**module**	Czochralski growth for mono-crystalline Si	Produces Si ingots. Improvements: Better control of crystal growth process through improved hot zone design, magnetic fields, recharging, computer simulations, preventing crucible contamination leading to higher quality crystals, larger ingots, and reduced material losses from cropping and shaping the ingot.	Material quality improvement; Process development; Tool development	Semiconductors	1970
**2**	**U_Si**	**module**	Multi-crystalline Si casting	Produces Si ingots. Improvements: Crucible coatings, seeding of silicon, gettering leading to higher quality and larger ingots, and reduced material losses from cropping and shaping the ingot.	Material quality improvement; Process development; Tool development	Metallurgy	1980
**23**	**U_Si**	**module**	LBD	Improvements in wafer, cell, module manufacturing by repetition of routine tasks	Non-innovation		
**24**	**p_Si**	**module**	Electric arc furnace	Produces ~98% pure metallurgical grade-Si (MG-Si) from silica. Improvements: Larger furnaces and improved energy efficiency.	Material quality improvement; Process development; Tool development	Metallurgy	1970
**25**	**p_Si**	**module**	Siemens process	Produces electronic grade silicon (polysilicon). Improvements: Increasing batch size, energy efficiency, improved handling	Material quality improvement; Process development; Tool development	Semiconductors	1970
**26**	**p_Si**	**module**	Fluidized Bed Reactor (FBR)	Produces electronic or solar grade silicon (polysilicon). More energy efficient than Siemens process, but hard to scale and more difficult to achieve electronic grade Si.	Material quality improvement; Process development; Tool development	Petroleum	1980
**1**	**p_Si**	**module**	Czochralski growth for mono-crystalline Si	Produces Si ingots. Improvements: Better control of crystal growth process through improved hot zone design, magnetic fields, recharging, computer simulations, preventing crucible contamination leading to higher quality crystals, larger ingots, and reduced material losses from cropping and shaping the ingot.	Material quality improvement; Process development; Tool development	Semiconductors	1970
**2**	**p_Si**	**module**	Multi-crystalline Si casting	Produces Si ingots. Improvements: Crucible coatings, seeding of silicon, gettering leading to higher quality and larger ingots, and reduced material losses from cropping and shaping the ingot.	Material quality improvement; Process development; Tool development	Metallurgy	1980
**27**	**p_Si**	**module**	LBD	Improvements in silicon purification process by repetition of routine tasks	Non-innovation		
**28**	**p_Si**	**module**	EOS	Larger polysilicon plants leading to lower unit manufacturing cost	Non-innovation		
**29**	**p_Si**	**module**	Other	Long-term contracts stabilizing prices	Non-innovation		
**30**	**p_Si**	**module**	Other	Supply-demand imbalances in the market (e.g. overcapacity and overproduction) might change polysilicon price even though production cost remain the same	Non-innovation		
**31**	**p_Si**	**module**	Other	Shifting production to locations with different electricity rates, labor rates and capital expenditures	Non-innovation		
**32**	**y**	**module**	Automated machinery	Processing steps (cell aligning, soldering, stringing, module assembly), loading/unloading between steps, and testing and inspection have been increasingly automated. Enabled by robotic material handling equipment, optical sensors, software to control/monitor devices and allow flexible operation. Reducing labor costs, while increasing yield, as thinner cells and heavy large items are handled more easily with automated machinery.	Automation; Tool development; Digitalization	Photovoltaics	1980
**33**	**y**	**module**	Preventing wafer edge and surface microcracks due to wafering	Fewer microcracks reduce wafer breakage. Achieved by using finer, more uniform SiC particles in SiC wire sawing; and by optimizing the sawing parameters such as speed and wire re-use in diamond wire sawing.	Process development; Tool development	Photovoltaics	1995

**34**	**y**	**module**	In-line process control	Optical inspection of incoming wafers for cracks and other defects using automated detection tools to reduce wafer breakage and ensure high quality cells. Electroluminescence imaging of cells and modules. Automatic optical inspection of cells in stringer.	Automation; Process development; Tool development; Digitalization	Photovoltaics	1980
**35**	**y**	**module**	LBD	Improvements in wafer, cell, module manufacturing by repetition of routine tasks	Non-innovation		
**1**	**A**	**module**	Czochralski growth for mono-crystalline Si	Produces Si ingots. Improvements: Better control of crystal growth process through improved hot zone design, magnetic fields, recharging, computer simulations, preventing crucible contamination leading to higher quality crystals, larger ingots, and reduced material losses from cropping and shaping the ingot.	Material quality improvement; Process development; Tool development	Semiconductors	1970
**2**	**A**	**module**	Multi-crystalline Si casting	Produces Si ingots. Improvements: Crucible coatings, seeding of silicon to obtain optimum grain sizes, gettering leading to higher quality and larger ingots, and reduced material losses from cropping and shaping the ingot.	Material quality improvement; Process development; Tool development	Metallurgy	1980
**36**	**A**	**module**	Overcoming limitations in handling large wafers	Enabled by automating and redesigning the processing and handling equipment. Standardization of tools allowed larger, uniform wafer areas while maintaining higher yields. Resulted in reduced cell and module manufacturing costs that show little area dependence.	Automation; Process development; Tool development	Semiconductors	1980
**37**	**A**	**module**	LBD	Improvements in wafer, cell, module manufacturing by repetition of routine tasks	Non-innovation		
**38**	**c**	**module**	Developments in silicon ingot manufacturing materials: crucible, graphite, argon	Crucibles with lower cost and longer life, crucible coatings; Cz hot zone designs that reduce argon and graphite consumption. These reduced cost per kg Si processed	Material quality improvement; Process development; Tool development	Semiconductors	1970
**39**	**c**	**module**	Developments in SiC wire sawing materials: slurry, wires, coolant, fixturing	Wires are used for cutting the ingots into wafers, and slurry facilitates the process. Slurry recycling was a key innovation, where the abrasive SiC particles and silicon debris are separated from polyethylene glycol.	Material quality improvement; Process development; Tool development	Semiconductors	1980
**40**	**c**	**module**	Developments in diamond wire sawing materials: wires, fixturing	Wires coated with diamond particles cut ingots into wafers. Compared to SiC slurry wire sawing, thinner and stronger wires, higher productivity and ease of recycling the kerf material	Material quality improvement; Process development; Tool development	Semiconductors	2011
**7**	**c**	**module**	Development and use of screenprinting of silver pastes	Silver paste forms metal contacts; allowed less doping with less capital intensive screenprinting equipment and increased throughput. Development of silver paste compositions and development of screenprinting process were key innovations.	Material quality improvement; Process development; Tool development	Electronics	1975
**8**	**c**	**module**	Development and use of screenprinting of aluminum pastes	Aluminum paste forms metal contacts at the back of the cell and creates back surface field (Al-BSF) architecture. Development of paste compositions and development of screenprinting process, especially the Al-BSF formation, were key innovations.	Material quality improvement; Process development; Tool development	Electronics	1975
**13**	**c**	**module**	Anti-reflective coated glass	Helps with light trapping improving power output. Innovation for PV was AR coating with reliability and durability needed for outdoor PV	Material quality improvement	Glass	2005
**41**	**c**	**module**	EVA laminate	Encapsulating the connected cells with low reflectivity allowing transfer of light. Over time the durability of PV EVA was improved Improvements to the additive formulation increased durability by preventing yellowing. There are other encapsulant materials, but EVA remains in widespread use mainly because of its good cost–performance ratio.	Material quality improvement	Electronics	1980

**16**	**c**	**module**	Qualification testing standards	IEC 61215 standards outline test procedures for design qualification mainly aiming for production quality control. Enabled balancing rigid quality requirements and allowed the development of low-cost modules. IEC 61730 outline PV module safety standards consistent with IEC 61215.	Standardization	Public institution	1993
**42**	**c**	**module**	Standards for module materials	IEC 62775, 62788, 62805 standards address polymeric packaging materials such as encapsulants, EVA, and TCO.	Standardization	Public institution	2010
**43**	**c**	**module**	Other	Easier access to adequate purity and low-cost chemicals (HF, HNO3, HCl)	Non-innovation		
**44**	**c**	**module**	Other	Dedicated PV glass production lines enabled lower glass cost	Non-innovation		
**45**	**c**	**module**	EOS	Bulk purchasing discounts reduce per-unit costs	Non-innovation		
**46**	**c**	**module**	Other	Facilities located near clusters of specialized material suppliers obtain cheaper materials	Non-innovation		
**47**	**K**	**module**	In-line processes	Tools with higher throughput due to higher speed; minimum handling, and lower breakage rate compared to batch processes. Conveyor belt length, width, speed, and the number of parallel lines can affect throughput.	Tool development; process development; Automation	Photovoltaics	1980
**12**	**K**	**module**	Tabber and stringer	Connecting solar cells. Key innovations include: Automation of stringing which reduced operation time and energy; machine vision for optical alignment of cells allowing narrower and more busbars reducing interconnect resistance, and detecting defects.	Automation; Tool development	Photovoltaics	1980
**48**	**K**	**module**	Production of Si ingots with larger cross-sectional area	Improvements in Cz process enabled better control of pull rate and temperature leading to larger diameters. Mc-Si ingots increased in size due faster crystal growth and other process improvements. These led to higher throughput and reduced power consumption.	Material quality improvement; Process development; Tool development	Semiconductors	1980
**49**	**K**	**module**	Other	Duplication of the same equipment to increase overall throughput	Non-innovation		
**48**	**p_0**	**module**	Production of Si ingots with larger cross-sectional area	Improvements in Cz process enabled better control of pull rate and temperature leading to larger diameters. Mc-Si ingots increased in size due faster crystal growth and other process improvements. These led to higher throughput and reduced power consumption.	Material quality improvement; Process development; Tool development	Semiconductors	1980
**6**	**p_0**	**module**	PECVD of SiNx for surface passivation	Depositing layer(s) for surface passivation and antireflective coating. PECVD of SiNx is a key innovation due to simplification of process by replacing multiple steps for passivation, AR coating, and paste control. Rear surface passivation is now emerging in PERC.	Material quality improvement; Process development; Tool development	Semiconductors	1983
**32**	**p_0**	**module**	Automated machinery	Processing steps (cell aligning, soldering, stringing, module assembly), loading/unloading between steps, and testing and inspection have been increasingly automated. Enabled by robotic material handling equipment, optical sensors, software to control/monitor devices and allow flexible operation. Reducing labor costs, while increasing yield, as thinner cells and heavy large items are handled more easily with automated machinery.	Automation; Tool development; Digitalization	Photovoltaics	1980
**50**	**p_0**	**module**	Turnkey manufacturing facilities	Includes equipment, process technology, and factory control. Enables fast entry to the market without much R&D experience. Often smaller factories and have higher capital costs	Automation; Tool development; standardization	Photovoltaics	2006
**51**	**p_0**	**module**	Standards for power performance testing	IEC 60904 series, IEC 60891, IEC 61853 focus on evaluating PV module power performance, increase the known reliability of the product and therefore decrease the cost of capital which the module manufacturer or a project developer is able to obtain.	Standardization	Public institution	1980
**52**	**p_0**	**module**	Other	Shifting production to locations with different electricity rates, labor rates and capital expenditures	Non-innovation		

**53**	**p_0**	**module**	LBD	Employee churning across facilities enables learning-by-doing and reduces labor costs	Non-innovation		
**54**	**p_0**	**module**	Other	Clustering of PV manufacturing supply chain in China	Non-innovation		
**55**	**p_inv_eta_inv**	**BOS**	Application specific integrated circuits (ASICs)	Microchips designed and optimized for specific applications (e.g. specific control strategies), reducing component counts (fewer individual devices, control cards)	Prefabrication/integration, Component design change	Semiconductors	1980
**56**	**p_inv_eta_inv**	**BOS**	High-frequency inverter designs	Combination of system architecture, circuit design changes and device innovations (use of MOSFETs, IGBTs) to increase inverter switching frequencies	Component design change	Semiconductors	2000
**57**	**p_inv_eta_inv**	**BOS**	Maximum power point tracking (MPPT)	Electric circuit designs and control strategies that adapt inverter resistance to maximize efficiency of power extraction from PV array (i.e. MPPT is the electronic equivalent to a tracker)	Component design change	Photovoltaics	1985
**58**	**p_inv_eta_inv**	**BOS**	Bi-directional inverters	Designs where all or most devices (e.g. switching elements) operate in both directions, enabling battery charging and discharging	Component design change	Semiconductors	1995
**59**	**p_inv_eta_inv**	**BOS**	Silicon insulated-gate bipolar transistors (Si IGBTs)	Semiconductor switching devices with increased switching frequencies	Component design change, Prefabrication/Integration	Power electronics	1980
**60**	**p_inv_eta_inv**	**BOS**	Silicon carbide (SiC) field effect transistors	Reduced losses (due to wide-bandgap material) allow for higher switching frequencies compared to Si IGBTs, which reduces the need for passive components like coils and capacitors, or allows smaller components. These effects reduce raw material usage and thus costs	Material quality improvement, Component design change, Prefabrication/Integration	Semiconductors	2010
**61**	**p_inv_eta_inv**	**BOS**	Gallium nitride (GaN) field effect transistors	Similar cost-reducing mechanisms as SiC devices; additional advantage is lateral structure which reduces stray inductances and parasitic resistances and therefore simplifies component packaging	Material quality improvement, Component design change, Prefabrication/Integration	Semiconductors	2015
**62**	**p_inv_eta_inv**	**BOS**	Thermal management strategies	Improved component layouts to increase heat dissipation into environment. Air cooling	Component design change	Semiconductors	2000
**63**	**p_inv_eta_inv**	**BOS**	Transformerless inverters	Change in national electric code (NEC) in 2010 that allowed transformerless inverters, which require less raw material (due to electronic instead of mechanical switching) and are therefore less costly	Component design change	Electronics	2010
**64**	**p_inv**	**BOS**	Automated optical inspection procedures	Automated visual circuit board inspection after every manufacturing step. Machine compares photograph of circuit board to reference data	Automation, Digitalization, Process development, tool development	Semiconductors	1980
**65**	**p_inv**	**BOS**	Soldering machines	Circuit board is moved through liquid solder paste to bond wire connections to circuit board	Automation, process development, tool development	Semiconductors	1980
**66**	**p_inv**	**BOS**	Printed circuit boards	Automated manufacturing of circuit boards instead of point-to-point construction	Automation, Digitalization, Process development, tool development	Electronics	1950
**67**	**p_inv**	**BOS**	Surface mount technology (SMT) component placement systems (also called pick-and-place or PNP machines)	Machines for placement of capacitors, coils, transistors, and other surface mounted devices on printed circuit boards	Automation, Digitalization, Process development, tool development	Semiconductors	1980
**68**	**p_inv_eta_inv**	**BOS**	String inverters	Reduced power losses due to centralized MPPT, reduced mismatch losses between modules, reduced string diode losses	Component design change, Architectural change	Photovoltaics	2000
**69**	**p_inv_eta_inv**	**BOS**	Microinverters	Simpler, faster installation. No extra installation for rapid shutdown requirement established by national electric code (NEC)	Component design change, Architectural change	Electronics	1994
**70**	**p_inv_eta_inv**	**BOS**	AC modules	Higher total per-Watt inverter costs	Prefabrication/integration, Architectural change	Photovoltaics	1994
**71**	**p_inv_eta_inv**	**BOS**	Multi-level inverter topologies	Increased range of inverter output voltages; allows smoother output waveforms, reducing harmonic distortions and voltage stress	Component design change	Electronics	1980
**72**	**p_inv_eta_inv**	**BOS**	Improved coil winding methods	Increased volume fraction used by windings to cut per-Watt material and space usage	Component design change	Electronics	1990

73	**p_inv_eta _inv**	**BOS**	High-efficiency inductors	Use of novel magnetic materials for inductor core (amorphous cores, ferrites, metal alloy powders) to reduce energy losses	Material quality, component design change	Semiconductors	2010
74	**p_inv_eta _inv**	**BOS**	Performance evaluation software	Simulations of thermal and electrical inverter behaviour instead of physical prototyping	Tool development, Digitalization	Semiconductors	2000
75	**p_inv_eta _inv**	**BOS**	IEEE 1547 standards	Series of standards that specify a set of universal criteria for the technically sound interconnection of distributed energy sources to the distribution grid; Consists of mandatory functional technical requirements (e.g. for equipment testing, as well as monitoring and control) and compliance options for equipment and equipment operation	Standardization	Public institution	2005
76	**p_inv_eta _inv**	**BOS**	Inverter performance test protocols	Series of standards for measuring inverter output characteristics (in particular inverter efficiency as a function of AC output power and DC voltage); one example is the California Energy Comissions's protocol; any inverter used in a CEC approved PV system must be tested by an independent lab to this protocol	Standardization, process development	Public institution	2000
77	**p_inv**	**BOS**	Modular inverter designs	Standardized smaller inverter units that can be assembled into larger inverters through series and parallel circuits; goal is to reduce the costs of customization for different PV applications, and to support the scale-up of the production of smaller units	Component design change, Architectural change	Electronics	1990
78	**p_inv_eta _inv**	**BOS**	Anti-islanding control	Circuit designs and control strategies to prevent power supply from PV system to grid during an outage; Innovations in early 2000s enabled pre-certification and low-cost implementation of anti-islanding controls (e.g. through software codes), thereby reducing inverter and interconnection costs	Prefabrication/Integrati on	Electronics	1990
79	**p_inv_eta _inv**	**BOS**	Integrated power modules	Replacement of multiple discrete components (conductors, transformer, filters) by one integrated module; an example is the AC filter-transformer module used in 2nd generation SMA inverters	Prefabrication/Integrati on	Electronics	2008
80	**p_inv_eta _inv**	**BOS**	Aluminum die-cast housing	Replacement of stainless steel housing with aluminum housing to improve specific heat capacity while reducing weight	Material quality improvement	Electronics	1990
81	**p_inv_eta _inv**	**BOS**	Printed circuit board (PCB) layout changes to enable large-scale manufacturing	Improvements in the choice of reference points (e.g. for component pick and place machines) to suit the manufacturing process	Non-innovation		
82	**p_inv_eta _inv**	**BOS**	Review of National Electric Code Article 690 (Solar Photovoltaic Systems)	Industry taskforce recommendations leading to review of code specifiying performance and installation (e.g. circuit design) requirements for PV systems; goal was simplification of code, adjustment to recent industry development;	Non-innovation		
83	**p_inv_eta _inv**	**BOS**	EOS in inverter factories	Reduced per-unit capex and opex due to larger output	Non-innovation		
84	**p_inv_eta _inv**	**BOS**	LBD in inverter factories	Manufacturing cost reductions due to incremental improvements in manufacturing steps resulting from repetition	Non-innovation		
85	**phi_a**	**BOS**	Wind tunnel testing of mounting systems	Experimentally testing structural stability of installer equipment allows for novel, reduced-material designs compared to previous, more conservative building codes and standards	Standardization, process development	Public institution	2012
86	**phi_a**	**BOS**	Module-integrated, railless mounting systems	Rails integrated into modules such that modules can be mounted directly to the roof	Prefabrication/integrati on	Photovoltaics	2007
87	**tau_s**	**BOS**	Bid preparation software platforms	Automated design of engineering and sales proposal, including financial analysis, system layout diagram, single-line drawing, contract preparation (e.g. PVBid); more comprehensive than remote shading analysis software (see below)	Tool development, Digitalization, automation	Construction	2010

**88**	**tau_s**	**BOS**	Remote site assessment software	Software for remote analysis site-specific conditions (shading, roof obstructions). An example is the use of satellite image instead of on-site measurements to create 2D image of building. Use of algorithm to construct 3D model and simulate shading. The result is a heat map of site-specific, shading-adjusted irradiance values	Tool development, Digitalization, automation	Photovoltaics	2010
**89**	**tau_s**	**BOS**	Building-integrated PV installation	PV integrated into building design from the beginning of design process to reduce time needed for PV-specific adjustments	Architectural change	Photovoltaics	1990
**90**	**tau_s**	**BOS**	Simplified zoning and planning laws	Simplify design requirements, thereby reducing design time	Legal innovation	Public institution	1970
**70**	**tau_s**	**BOS**	AC modules	Eliminating DC circuit reduces design time	Prefabrication/integration, Architectural change	Photovoltaics	1994
**77**	**tau_s**	**BOS**	Modular inverter designs	Standardized smaller inverter units that can be assembled into larger inverters through series and parallel circuits; goal is to reduce the costs of customization for different PV applications, and to support the scale-up of the production of smaller units	Component design change	Electronics	1990
**91**	**tau_s**	**BOS**	Plug-and-play PV systems	Pre-configured electrical connections that require no manual field wiring and reduce overall number of connections that need to be made on-site. System standardization will likely also reduce system design time (particularly in extreme forms of 'off-the-shelf' plug-and-play designs).	Prefabrication/integration, Architectural change	Photovoltaics, Public institution	2000
**92**	**tau_s**	**BOS**	LBD	Faster system design through incremental improvements resulting from repetition of design tasks	Non-innovation		
**93**	**tau_m**	**BOS**	Integrated mounting systems ("plug-and-play" mounting, "solar platforms")	Prefabricated mounting systems reduce component count and need for tools, thereby reducing on-site installation time (fewer and simpler steps)	Prefabrication/integration, Architectural change	Photovoltaics	2010
**86**	**tau_m**	**BOS**	Module-integrated, railless mounting systems	Rails integrated into modules such that modules can be mounted directly to the roof	Prefabrication/integration	Photovoltaics	2007
**94**	**tau_m**	**BOS**	Integrated hook and clamp solutions (direct attachment mounting)	Integrate standard grounding features in clamp. No need to install grounding separately	Prefabrication/integration	Construction	1995
**89**	**tau_m**	**BOS**	Building-integrated PV installation	PV integrated into building design from the beginning of design process to reduce time needed for PV-specific installation	Architectural change	Photovoltaics	1990
**95**	**tau_m**	**BOS**	Non-penetrating mounting systems	Ballasted support system for fixing PV panels on roof using strategically placed weights (e.g. cinder blocks) to achieve stability without bolting rails down by penetrating roof membrane (often used for low-tilt roofs, or when roof too old to be penetrated; not suitable in high-wind areas)	Prefabrication/integration	Construction	1990
**91**	**tau_m**	**BOS**	Plug-and-play PV systems	Pre-configured electrical connections that require no manual field wiring and reduce overall number of connections that need to be made on-site. System standardization will likely also reduce system design time (particularly in extreme forms of 'off-the-shelf' plug-and-play designs).	Prefabrication/integration, Architectural change	Photovoltaics, Public institution	2000
**96**	**tau_m**	**BOS**	LBD	Faster installation through incremental process efficiency improvements resulting from repetition of installation tasks; LBD can result in lower idle time of workers on-site, better organization of crew schedules etc.	Non-innovation		
**69**	**tau_e**	**BOS**	Microinverters	Simpler, faster installation. No extra installation for rapid shutdown requirement established by national electric code (NEC)	Component design change, Architectural change	Electronics	1994
**70**	**tau_e**	**BOS**	AC modules	Simpler, faster installation because microinverter (AC modules) or DC optimizer (smart modules) already integrated into module. No extra installation for NEC rapid shutdown requirement (see above)	Prefabrication/integration, Architectural change	Photovoltaics	1994

**97**	**tau_e**	**BOS**	Easy-to-separate PV cables	Faster, safer installation because co-extruded cables can be separated using fingers instead of cutter. Positive and negative conductor can nevertheless be transported on single spool	Prefabrication/integration	Electronics	2010
**98**	**tau_e**	**BOS**	Y-connectors	Connectors with one input and two outputs allow simplified ("ready-to-plug") parallel circuit connections	Non-innovation		
**91**	**tau_e**	**BOS**	Plug-and-play PV systems	Pre-configured electrical connections that require no manual field wiring and reduce overall number of connections that need to be made on-site	Prefabrication/integration, Architectural change	Photovoltaics, Public institution	2000
**99**	**tau_e**	**BOS**	Wireless inverter configuration tools	Apps for simplified inverter activation and broadcasting of firmware updates through Wifi/Bluetooth	Digitalization, tool development	Electronics	2005
**100**	**tau_e**	**BOS**	DC optimizers	DC-DC-converters installed with each individual module; converters adjust their output voltage to match module output current to string current, thereby maximizing conversion efficiency through adjustment of (i.e. maximum-power-point tracking at the module level)	Component design change	Electronics	2009
**101**	**tau_e**	**BOS**	LBD	Faster installation through incremental improvements resulting from repetition of installation tasks	Non-innovation		
**102**	**tau_PII**	**BOS**	Full online permitting	Enables completion of all aspects of the permit process (application submittal, plan review, fee payment, delivery of approved permits via email or a website) online, often faster than before	Digitalization	Petroleum	2010
**103**	**tau_PII**	**BOS**	Template for single line diagram	Template for single line diagram that replaced customized single line diagrams	Standardization	Photovoltaics	2012
**104**	**tau_PII**	**BOS**	Cross-training programs for permit staff	Cross-training programs for electrical and building inspectors (goal: one site visit instead of two)	Non-innovation	Public institution	2010
**105**	**tau_PII**	**BOS**	Automated engineering review of grid interconnection	Automated screening system aggregates equipment information and site specifications provided via application portal, distribution-feeder information, and billing information. This information is then linked to built-in calculations to automatically complete initial review screens (e.g. whether interconnection will exceed acceptable transformer loads)	Tool, Digitalization, Automation	Electronics	2010
**106**	**tau_PII**	**BOS**	Solar permit application checklist	Compact summary of technical requirements for homeowners (the innovation is to translate experiences from previous questions into effective information)	Non-innovation	Electronics	2000
**107**	**tau_PII**	**BOS**	Software applications to improve interconnection workflow management for utility	Software that consolidates internal management and processing of interconnections under different interconnection rules. Simplifies document retention and retrieval by enabling departments within a company to interact with a common database	Automation, Digitalization, tool development	Electronics	2010
**108**	**tau_PII**	**BOS**	Fast track permitting	Expedited permitting for small-scale, standard systems	Standardization, process development	Public institution	2010
**75**	**tau_PII**	**BOS**	IEEE 1547 standards	Series of standards that specify a set of universal criteria for the technically sound interconnection of distributed energy sources to the distribution grid; Consists of mandatory functional technical requirements (e.g. for equipment testing, as well as monitoring and control) and compliance options for equipment and equipment operation	Standardization	Public institution	2005
**78**	**tau_PII**	**BOS**	Anti-islanding control	Circuit designs and control strategies to prevent power supply from PV system to grid during an outage; Innovations in early 2000s enabled pre-certification and low-cost implementation of anti-islanding controls (e.g. through software codes), thereby reducing inverter and interconnection costs	Prefabrication/integration, Architectural change	Electronics	1990
**91**	**tau_PII**	**BOS**	Plug-and-play PV systems	Pre-configured electrical connections that require no manual field wiring and reduce overall number of connections that need to be made on-site	Prefabrication/integration, Architectural change	Photovoltaics, Public institution	2000

**76**	**tau_PII**	**BOS**	Inverter performance test protocols	Series of standards for measuring inverter output characteristics (in particular inverter efficiency as a function of AC output power and DC voltage); one example is the California Energy Comissions's protocol; any inverter used in a CEC approved PV system must be tested by an independent lab to this protocol	Standardization	Public institution	2000
**109**	**tau_PII**	**BOS**	Policy regulation	New building codes that made it easier to install and permit PV (e.g. California solar PV guidebook)	Non-innovation		
**110**	**tau_PII**	**BOS**	Transparent permitting and interconnection requirements	Improved public access (e.g. online access) to information on requirements for PV permits	Non-innovation		
**111**	**tau_PII**	**BOS**	Online interconnection application and submission	One single point of entry for applications that previously came via mail, email, fax	Non-innovation		
**112**	**tau_PII**	**BOS**	LBD	Faster permitting due to repetition and accumulating experience in permitting office, electrical inspection etc.	Non-innovation		
**113**	**p_sc**	**BOS**	E-commerce marketplaces	Enables smaller firms to gain centralized access to a larger pool of products. Could in future allow aggregating orders by multiple small installers to benefit from bulk purchase prices	Non-innovation		
**114**	**K_inv**	**BOS**	Oversizing	Increasing the ratio of module dc power to inverter ac power to a number larger than one to increase energy yield	Architectural change	Public institution	2010
**115**	**K_inv**	**BOS**	LBD in module factories	Inherited from module because lower cost modules encouraged oversizing	Non-innovation		
**116**	**K_inv**	**BOS**	EOS in module factories	Inherited from module because lower cost modules encouraged oversizing	Non-innovation		
**117**	**eta_w**	**BOS**	LBD	Incremental improvements in wire and cable layouts resulting from repeated system design	Non-innovation		
**118**	**p_w**	**BOS**	EOS	Bulk purchases of wires and cables to reduce per-unit costs	Non-innovation		
**119**	**p_w**	**BOS**	Other	Drivers of changes in cable and wire prices inside or outside the boundary of the PV industry - uncertain drivers from perspective of PV industry	Non-innovation		
**120**	**p_a**	**BOS**	Other	Drivers of changes in commodity prices inside or outside the boundary of the PV industry - uncertain drivers from perspective of PV industry	Non-innovation		
**121**	**w_e**	**BOS**	Other	Drivers of wage changes inside or outside the boundary of the PV industry - uncertain drivers from perspective of PV industry	Non-innovation		
**122**	**w_m**	**BOS**	Other	Drivers of wage changes inside or outside the boundary of the PV industry - uncertain drivers from perspective of PV industry	Non-innovation		
**123**	**w_s**	**BOS**	Other	Drivers of wage changes inside or outside the boundary of the PV industry - uncertain drivers from perspective of PV industry	Non-innovation		
**124**	**w_PII**	**BOS**	Other	Drivers of wage changes inside or outside the boundary of the PV industry - uncertain drivers from perspective of PV industry	Non-innovation		
**125**	**p_r**	**BOS**	EOS	Bulk purchases of racking systems by installers to reduce per-unit costs	Non-innovation		
**126**	**p_r**	**BOS**	Pricing strategy	Firm-level pricing decisions	Non-innovation		
**127**	**p_oe**	**BOS**	EOS	Bulk purchases of non-inverter electrical hardware (meters, monitors) to reduce per-unit costs	Non-innovation		
**128**	**p_oe**	**BOS**	Other	Drivers of hardware cost change outside the PV industry	Non-innovation		
**129**	**c_PII**	**BOS**	Policy regulation	Lowering permitting fees	Non-innovation		
**130**	**p_op**	**BOS**	EOS	Economies of scale at the firm level reducing overhead per Watt installed	Non-innovation		
**69**	**eta_w**	**BOS**	Microinverters	Simpler, faster installation. No extra installation for rapid shutdown requirement established by national electric code (NEC)	Component design change, Architectural change	Electronics	1994
**70**	**eta_w**	**BOS**	AC modules	Simpler, faster installation because microinverter (AC modules) or DC optimizer (smart modules) already integrated into module. No extra installation for NEC rapid shutdown requirement (see above)	Prefabrication/integration, Architectural change	Photovoltaics	1994

S1 Appendix. Data and methods used for Example 1: Estimating the cost impacts of silicon carbide wire sawing

We estimate the cost impact of switching to wire sawing from internal diameter (ID) saws. To do this we identify which variables of the module cost equation were affected by the switch. Based on a literature review, we determine that mainly (1) silicon utilization and (2) throughput (i.e. our plant size variable which is measured in number of modules produced in a given year) were impacted by the introduction of wire sawing. (These variables are circled in dark green in the cost equation shown in S1 Figure.) Since wire sawing was introduced in the 1980s, we model its cost impacts based on the state of the technology around that time. We first populate our module cost equation with data for 1980 (see S2 Table). We then change the silicon utilization and plant size variables in the direction indicated by the literature. We estimate the impact of these changes on module cost and the contribution of impacted variables to cost change using our cost change model [1].

Silicon loss during wafering was reduced by about 30% due to wire saws [4], from 50% to 35%. Assuming that silicon thickness remained at 500 μm, our silicon usage variable (which is calculated as silicon thickness divided by silicon utilization) is reduced from 0.25 cm to 0.14 cm. Plant size increased by 30% owing to more wafers per inch of ingot [5]. Assuming that efficiency and thickness are the same as before, this translates into an overall plant size increase of 30%. Overall, these changes lead to a cost decrease from 29 \$/W in 1980 to 24 \$/W (2015 USD).

S1 Fig. Module cost equation. The variables affected by wire sawing are circled. Dark green circles indicate the variables whose cost impacts were analyzed in S1 Appendix and main text section 3.6; light green circles indicate the other variables that were potentially affected by the introduction of wire sawing.

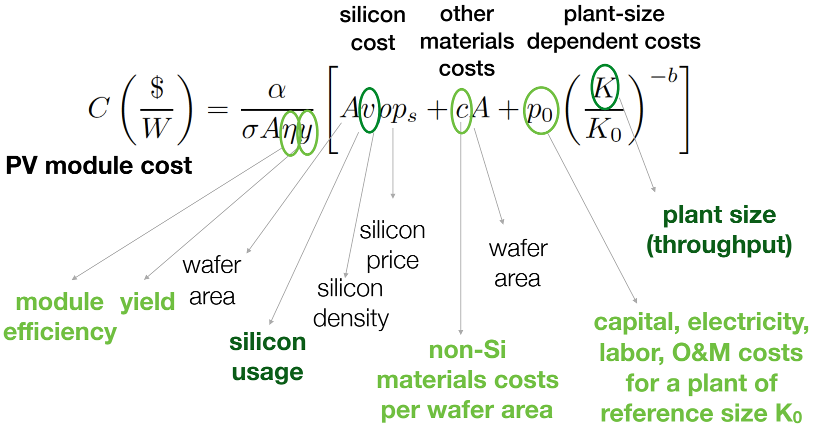

Variable	Unit	Before wire sawing (1980 values)	After wire sawing
Plant size (throughput) (K)	modules/year	1 MW/year = about 17200 modules/year	30% increase: 1.3 MW = about 22,300 modules/year. "30% increase in production volume owing to more wafers per inch of ingot" [5]. Assuming that efficiency and thickness are the same as before, this translates into an overall throughput increase of 30%.
Silicon thickness (t)	μm	500 μm	same as before
Silicon utilization (U)	unitless	20%. Losses during ingot growth and cutting (30%), and wafering (50% for ID saws [3]) are accounted for.	35%. Wafering losses are reduced by about 30% due to wire saws [4], from 50% to 35%. Ingot growth and shaping losses are assumed to be the same as before.
Silicon usage ($v = t/U$)	cm	0.25	0.14
Module efficiency (η)	unitless	8%	same as before
Polysilicon price (p_s)	2015 $/kg	126	same as before
Wafer area (A)	cm^2	90	same as before
Yield (y)	unitless	75%	same as before
Non-Si materials costs per wafer area (c)	2015 $/cm^2	0.062	same as before
Capital, labor, O&M, electricity costs (p_0)	2015 $	1.32	same as before

S2 Table. Data for cost equation before wire sawing (1980 values) and after wire sawing. Data have been collected from multiple sources; only the mean values used to populate the equation are shown in this table. c and p_0 are calculated using various other data. Note that the scaling factor, b, in the equation is 0.27.

S2 Figure shows the contribution of the affected variables to module cost change. (As a reminder, we call the changes in the cost equation variables 'low-level mechanisms' of cost change.) We observe that by reducing the silicon losses during wafering by 30%, wire sawing reduces the module cost by 4.66 \$/W (2015 USD). (ΔC_v = -4.66 \$/W). By increasing the plant size by 30%, it reduces the module cost by 0.62 \$/W (2015 USD). (ΔC_K = -0.62 \$/W). Overall, these lead to a cost decrease from 29 \$/W to 24 \$/W (2015 USD). Silicon usage reduction contributed 88% of this cost change, while increased plant size contributed 12%.

S2 Fig. Low-level mechanisms of cost change due to a switch to wire sawing from internal diameter saws for wafering. Changes in silicon usage and plant size variables lead to a cost decrease from 29 \$/W to 24 \$/W (2015 USD) in total. About 90% of this cost change came from a reduction in silicon usage.

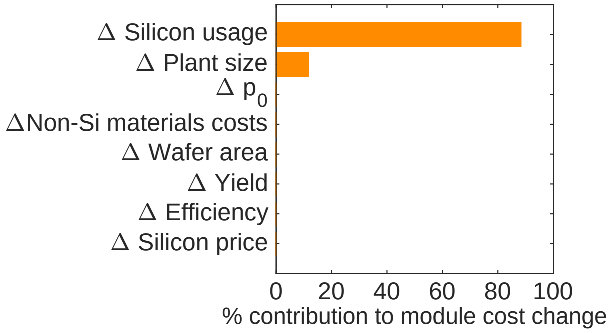

S2 Appendix. Data and methods used for Example 2: Attributing the non-silicon materials cost reductions to innovations

This section shows the data and methods used to estimate the cost effects of the innovations affecting the variable c (non-silicon materials costs.) 'Non-silicon materials costs' is an aggregate variable which is the sum of the costs of materials needed to produce silicon ingots such as crucibles and argon, wire sawing materials, silver and aluminum pastes and associated screenprinting materials, anti-reflective coated glass, and other cell and module materials.

For this example, we are using a cost equation for PV modules rather than PV systems. The module cost equation is given as part of Eq. 1 and the variable definitions are provided in Section 2.1.

$$C\left(\frac{\$}{W}\right) = \frac{\alpha}{\sigma A \eta y}\left[A v \rho p_s + cA + p_0\left(\frac{K}{K_0}\right)^{-b}\right] \tag{A-1}$$

We obtain the data on non-silicon material costs for years 2010 and 2018 from [6, 7]. (However, the cost of glass in 2010 could not be determined so we will use the cost data for glass in 2012 in [2] as an approximation.) The models in [6, 7] report costs in \$/W, rather than the unit for c, which is \$/cm^2, referring to non-silicon material costs per wafer area. Therefore, in order to obtain non-silicon material costs in \$/W, c must be multiplied by the term $\alpha/(\sigma\eta y)$. For the purposes of this analysis we create a new variable c_W as defined below, which represents non-silicon materials costs measured in \$/W:

$$c_W = c\frac{\alpha}{\sigma\eta y} \tag{A-2}$$

S3 Figure shows the the value of c_W (cost of non-silicon materials in \$/W) in 2010, then displays the cost reductions that occurred in the subcomponent material categories mentioned above up through 2018, and then finally shows the the value of c_W (cost of non-silicon materials \$/W) in 2018. From this figure, it is apparent that most of the cost reductions occur in wire sawing materials, silver paste metallization, and ARC glass.

If we want to identify the portion of these cost changes which can be attributed to reductions in c, any changes in the $\alpha/(\sigma\eta y)$ term over this period must first be evaluated and removed. To do this, we assume that α remains constant over the 2010-2018 period [1]. Changes in efficiency (η) are documented as 14.4% in 2010 in [6] and as 18% in 2018 in [7]. For y, we assume a 2010 value of 94% based on 2012 data in [1], and extrapolate a value of 97% for 2018. This results in approximately a 20% reduction in the $\alpha/(\sigma\eta y)$ term over the 2010-2018 time period, whereas c_W(non-silicon material costs in \$/W) decreased by 72% over the same time period. Therefore, we can estimate that innovations specific to c reduced c by 65% over 2010-2018.

Next we determine which innovations and non-innovations affect which subcomponent material category of c. Then we apply the rough estimate that the cost reduction caused by each subcomponent material category is equally distributed across the innovations and non-innovations affecting that category as shown in Figure 6 The resulting contributions of each of the innovations and non-innovations to the overall reduction of costs are shown in the main text (Section 3.6 and Fig. 6).

S3 Fig. Non-silicon materials costs. Reductions in non-silicon materials costs \$/W from 2010 to 2018.

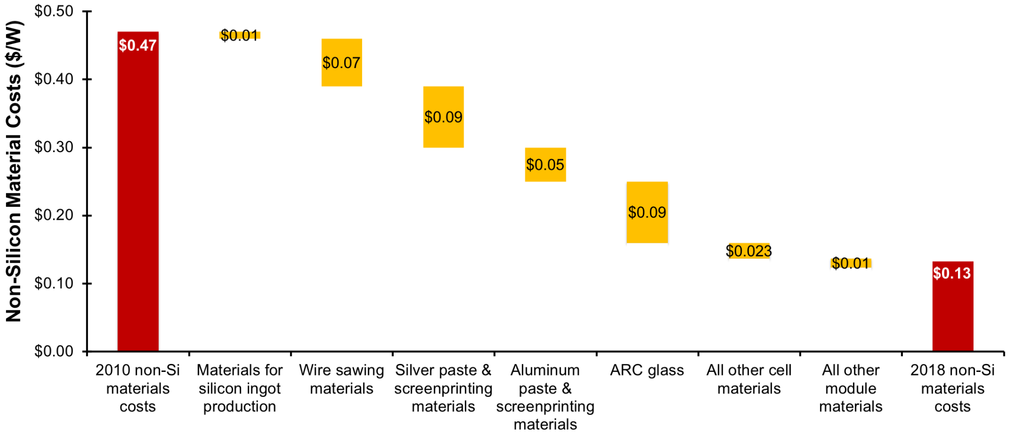

S3 Appendix. Expert feedback on the innovations table

The innovations table was initially developed after a literature review, and then sent to experts in the PV industry to solicit feedback on a) the comprehensiveness of the table in terms of covering key PV innovations and b) the assignment of innovations to cost variables. The feedback forms sent to the experts for the module variables and balance-of-system variables are shown at the end of this section (module variables, pp. 19-27; BOS variables, pp. 28-34).

For modules, the feedback suggested that we had a comprehensive list of innovations in our initial table. Some of the innovations that were suggested by the experts as potential additions to the table included larger Siemens furnaces, larger crucible and ingot sizes, Cz growth using multiple charges, decreased glass cost due to increased volume justifying dedicated PV glass production lines, silver paste with reduced silver content, transition to higher efficiency cell designs, particularly PERC, AR coatings for front glass of the module has improved the power output. We added new innovations based on the experts' feedback, such as 'automation of stringing','passivated emitter and rear cell (PERC)' and 'AR coatings for glass'.

We also updated a few of the module innovations based on the feedback. For example, instead of 'overcoming limitations in handling and processing thinner wafer', we now have 'contactless soldering of ribbons.' Finally, we removed some of the innovations from our initial table since the comments indicated that the effects of these may not be significant.

We also discussed the industry origins of innovations to set the stage for our more detailed analysis. Comments indicated that early PV production borrowed heavily on the semiconductor industry for both tools and processes. We discussed specific innovations such as screen printing tools, which came mainly from the PC board industry. As production volumes grew, dedicated PV tool suppliers started producing specialized tools were developed with an emphasis on wafer throughput. This confirmed our prior knowledge about the contributions of the semiconductor industry to PV.

The experts also pointed us to contributions to innovations from the public and private sector. For example, the development of the wire saw, a critical enabler of low cost, was first explored by government organizations (JPL in this case), and then the actual commercial development was done by companies that developed wire saw equipment, such as HCT. Beginning in the 1990s, as the industry grew, significant private R&D started to emerge. Experts mentioned that since approximately the year 2000, the innovation to advance the mainstream technology is being done by the private sector, while public R&D plays a supporting role for the conventional PV technology based on silicon wafer, and focuses on other PV technologies and innovations that might have an impact in the future.

To set the stage for our more detailed analysis on the time stamps for innovations, we also had a general discussion on the timelines for different types of innovations. Experts suggested that innovations focused on developing processes until mid-1990s, developing equipment from mid-1990s to approximately 2010, and commoditization has been prevalent since then. At the time of this feedback, our typology hadn't been developed yet. However, we ended up including process and tool development as two types of innovations in our final innovations typology.

The comments on the non-innovation drivers included some of the possible reasons that might have enabled cost reduction in China. Experts mentioned that 'China' likely meaning 'Chinese' policies but the experts did not specify further supported supply chain capacity expansion to a large extent in the more recent years. Factors such as collaboration among different stakeholders in the supply chain, employee churning, convenient communication channels, manufacturing clusters were counted among the potential reasons for cost decline in China.

For BOS innovations, we received feedback from two external reviewers. While the feedback has been primarily positive with regards to the comprehensiveness of the table, and the matching of innovations to variables, several comments have motivated refinements of the table:

1. Expansion of the inverter-related section of the innovations table by six items (innovations 57-62, see S1 Appendix)
2. Recognition of indirect benefits of BOS innovations that affect dimensions of technology performance other than installed costs (reliability, safety, monitoring functions)
3. Additional information on the origin of innovations (e.g., PV industry vs. electronics industry)
4. Better understanding of the role of country-specific regulations in shaping inverter trends

We discuss each point in more detail below.

Expansion of inverter innovations list. One of our reviewers identified five innovations that have affected inverter prices and inverter efficiency and were not included so far. The emergence of different inverter families (string inverters, module-level inverters) was also highlighted as a separate innovation by another reviewer. We list the additional innovations in our innovations table (S1 Appendix. As one example, multilevel inverter topologies increase inverter efficiencies and, as the reviewer framed it, can also increase the potential for cost reduction. Instead of generating an output waveform with two voltage levels (two-level inverter architecture), multilevel inverters use semiconductor power switches along with several internal lower voltage dc levels to synthesize a smoother output waveform. This change reduces harmonic distortions and voltage stresses on the power switches, thereby reducing filtering needs and associated commodity usage and component counts, while also increasing inverter efficiency. Due to the higher use of semiconductor materials instead of metals, the reviewer also thinks that investments in multi-level inverter R&D can make inverters more amenable to the more steady cost reductions observed in semiconductor manufacturing (as opposed to the volatile price trends observed for aluminum, iron, and copper). Sustainability is another driver of the trend towards reduced commodity usage in inverters. The full updated BOS innovations table is given in S1 Appendix.

Recognition of indirect benefits of BOS innovations. Two reviewers commented that some innovations likely resulted in technology performance improvements on other dimensions than cost. Inverter efficiency increases, for example, have resulted in smaller devices, reducing costs per Watt but also space requirements. The development of wall-mounted inverters with reduced spatial footprint was a consequence. A second example is the use of improved inspection techniques for inverter quality control. According to a reviewer, this change did not affect upfront inverter costs, but operations and maintenance costs as well as customer satisfaction. A third example highlighted by a reviewer is the role of software in reducing the cost of PV system asset management, and in enhancing reliability through increased visibility of failures. Innovations in this area do not affect the variables in the BOS cost equation, which represents upfront costs, but they would affect operations and maintenance costs.

Additional information on the origin of innovations. Asked whether inverter-related innovations originated in the PV industry or in the electronics industry, one reviewer highlighted the role of PV-focused efforts in developing multilevel inverter topologies, wide bandgap semiconductor inverters, and transformerless inverters. In other areas, however, (digital signal processing, microcontrollers, field programmable gate arrays), the PV industry benefited from innovations made in other industries. These knowledge spillovers were, according to the reviewer, critical in the transition from analog to digital inverter control.

Better understanding of the role of country-specific regulations in shaping inverter trends. One reviewer noted that the recent trend toward module-level power electronics (e.g., microinverters) is related to the emphasis placed on safety in the U.S., and therefore country-specific. High DC voltages in PV systems with central or string inverters are considered a source of danger particularly for fire fighters that need to act rapidly. Module-level power electronics can reduce this risk by converting DC to AC power at the module-level, but are more expensive on a $/W basis.

Feedback forms. The following pages show the feedback forms sent to experts for the module variables (pp. 19-27) and BOS variables (pp.28-34).

PV module innovations July 2018

Background. We are evaluating the mechanisms driving photovoltaic (PV) system cost reductions, delving deeply into specific technological innovations that have occurred in the past, and the policies that have encouraged them. This project has three main objectives: (1) identify mechanisms of cost reductions in PV systems (both 'low-level' (e.g. conversion efficiency improvement) and 'high-level' (e.g. R&D efforts)); (2) identify specific innovations that enabled the low-level mechanisms of cost reduction; (3) understand how public policies supporting PV deployment and R&D contributed to PV's cost improvement. Currently we are focusing on objective 2. Our immediate goals are to identify the innovations that were most instrumental in reducing costs since 1980, and better understand how these innovations affected costs. The project as a whole examines both module and balance-of-system (BOS) cost changes and innovations. In this expert feedback request we focus on PV modules.

Your input. Please have a look at the material starting on page 2 about PV module costs and innovations. On page 2, we describe a cost model that we have developed for PV module production. On page 3, we then list the variables of this cost model in a table together with historical innovations that may have caused each variable to improve. We would appreciate your suggestions on how to improve this table.

In particular, we would appreciate your input on

(1) whether our list covers the most important innovations, historically or on-going;
(2) whether the variables that each innovation affects are identified correctly;
(3) whether the innovation took place in the context of public R&D or private R&D;
(4) whether the innovation originated in the PV industry or somewhere else;
(5) whether the innovation involved tradeoffs, improving one variable at the expense of another (for instance, screenprinting increased throughput but came with an efficiency loss.);
(6) the estimated time range for the innovation occurred.

You can respond to questions (1) and (2) on page 7. You can respond to questions (3)-(6) either on page 7, or directly in the innovations table in the empty cells next to each innovation.

Description of innovations table. We focus on innovations that may have affected the cost of conventional multi-and monocrystalline silicon-based PV modules in the period 1980-2017. In the first column we list key variables that determine a PV module's cost. In the second column we assign a number to each innovation. Feel free to use these numbers to refer to innovations in your answers on page 7. In the third column we list innovations that may have affected each variable and potentially led to cost reduction. An appendix table on page 8 provides brief additional explanations of the innovations.

MODULE COST EQUATION

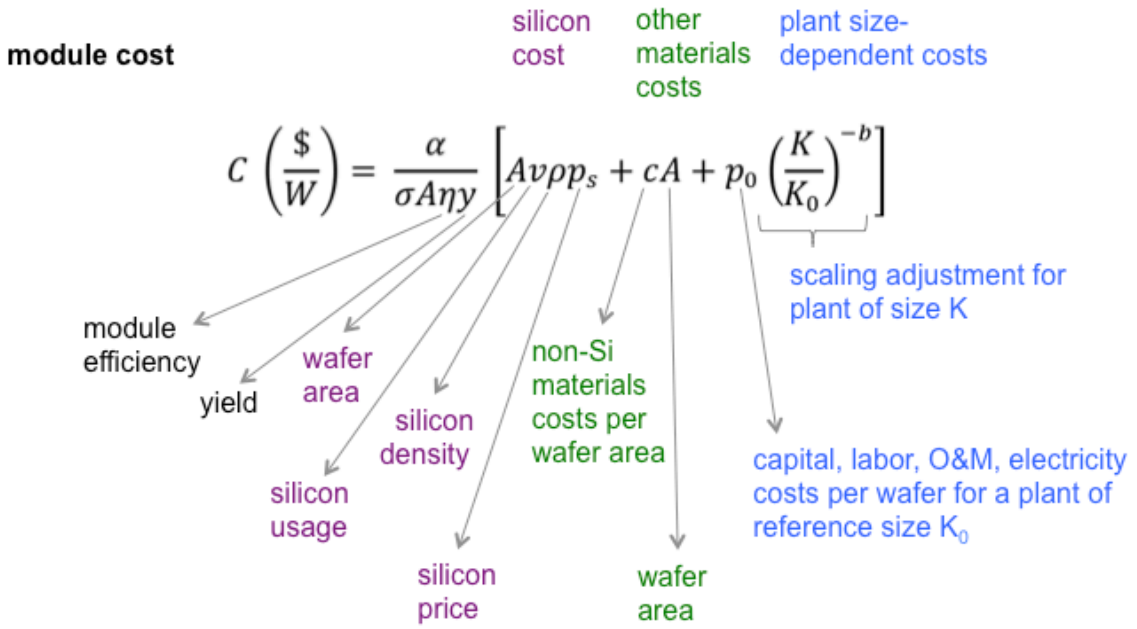

MODULE COST VARIABLES

A	Wafer area (cm^2)
η	Module efficiency (unitless)
y	Yield (unitless)
v	Silicon usage (cm) (calculated as the wafer thickness divided by silicon utilization)
p_s	Silicon (polysilicon) price ($/kg)
c	Non-silicon materials costs per wafer area ($/$cm^2$)
p_0	Capital, labor, O&M, electricity costs per wafer (which we model as plant-size dependent) for a plant of reference size K_0 ($)
K	Plant size (modules/year)

The remaining are parameters defined as following:

α	Fraction of module area used by cells (0.89)
σ	Solar constant (0.1 W/cm^2)
ρ	Density of silicon (2.33 g/cm^3)
b	Scaling factor (0.27)
K_0	Number of modules manufactured in a year in a 1000 MW plant (the typical plant size value for 2012).

INNOVATIONS TABLE

Variables	Innovation number	Innovations	Origins: Did the innovation take place in the context of public R&D (universities, national labs) or private R&D (industry)?	Origins: Was this innovation specific to PV, or did it come from another industry?	Tradeoffs: Did this innovation introduce any tradeoffs or penalties? If so what were they? (e.g. Screen printing increased throughput but came with an efficiency penalty.)	Time: What is an estimated time range for the innovation?
η Module efficiency (unitless)	***Wafer level***					
	1	Electric arc furnace				
	2	Siemens process, FBR, UMG-Si				
	3	Czochralski growth for mono-crystalline Si				
	4	Multi-crystalline Si casting				
	Cell level					
	5	In-line wafer characterization				
	6	Wet-chemical etching for texturing				
	7	Belt furnace, tube diffusion, or ion implantation for P diffusion				
	8	PECVD or ALD for surface passivation				
	9	Screenprinting for metal contact formation				
	Module level					
	10	Reducing variance in cell quality				
	11	Tabbing and stringing				
	12	Lamination				
		Other innovations?				
t Si wafer thickness (μm)	13	Early wafering methods such as inner diameter blade saw				
	14	SiC wire sawing				
	15	Diamond wire sawing				
	16	Overcoming limitations in handling and processing thinner wafers				
		Other innovations?				

Variables	Innovation number	Innovations	Origins: Did the innovation take place in the context of public R&D (universities, national labs) or private R&D (industry)?	Origins: Was this innovation specific to PV, or did it come from another industry?	Tradeoffs: Did this innovation introduce any tradeoffs or penalties? If so what were they? (e.g. Screen printing increased throughput but came with an efficiency penalty.)	Time: What is an estimated time range for the innovation?
U **Silicon utilization during ingot cutting, wafering etc. (unitless)**	17	Wafering methods: wire-sawing, inner diameter blade saw				
	18	Production of ingots with larger cross-sectional area				
		Other innovations?				
p_s **Si price ($/kg)**	19	Various innovations to tools and materials in MG-Si production and polysilicon production processes				
		Other innovations?				
y **Yield (unitless)**	20	Automated transfer machinery				
	21	Preventing wafer surface microcracks				
	22	Reducing the number of and improving the wafer handling steps				
	23	In-line characterization of wafers				
	24	Laser-based processes as opposed to wet-bench				
	25	Integration of module assembly processes				
		Other innovations?				

Variables	Innovation number	Innovations	Origins: Did the innovation take place in the context of public R&D (universities, national labs) or private R&D (industry)?	Origins: Was this innovation specific to PV, or did it come from another industry?	Tradeoffs: Did this innovation introduce any tradeoffs or penalties? If so what were they? (e.g. Screenprinting increased throughput but came with an efficiency penalty.)	Time: What is an estimated time range for the innovation?
A **Wafer area (cm2)**	26	Better control in Si crystallization				
	27	Overcoming limitations in handling large wafers				
	28	Overcoming limitations due to higher series resistance in larger wafers				
		Other innovations?				
α **Packing density (unitless)**	29	Quasisquare and square shapes of multicystalline wafers				
		Other innovations?				
c **Non-Si materials costs ($/cm^2)**	30	Development and use of wafer materials such as crucible, argon				
	31	Wire slurry, wires, coolant, fixturing for wire-sawing				
	32	Silver paste (screenprinted to form front contacts)				
	33	Aluminum (screenprinted on back surface)				
	34	Development and use of other cell materials such as chemicals, screens				
	35	Development and use of module materials such as encapsulants (e.g.EVA), adhesives, backsheet, tempered low-iron antireflective glass, Al frame, silicone sealant, J-box				
		Other innovations?				

Variables	Innovation number	Innovations	Origins: Did the innovation take place in the context of public R&D (universities, national labs) or private R&D (industry)?	Origins: Was this innovation specific to PV, or did it come from another industry?	Tradeoffs: Did this innovation introduce any tradeoffs or penalties? If so what were they? (e.g. Screenprinting increased throughput but came with an efficiency penalty.)	Time: What is an estimated time range for the innovation?
K **Plant size (modules/ year or MW/yr)**	36	Tools with higher throughput				
	37	Turnkey manufacturing facilities				
	38	Developed equipment manufacturers				
	39	Developments in material supply industries				
		Other innovations?				
p0 **Capital, labor, O&M, electricity costs ($)**	40	Larger silicon ingot sizes				
	41	Faster processing techniques				
	42	Simplified processes				
	43	Lower-temperature processes				
	44	Laser-based processes				
	45	Replacing vacuum with non-vacuum processes				
	46	Increased labor productivity				
	47	Improvements in manufacturing technologies and equipment				
	48	Increased automation				
		Other innovations?				

QUESTIONS

(1) Does our list cover the most important innovations, historically or ongoing?

(2) Did we correctly identify the variables that each innovation affects? If not, which revisions would you suggest?

[You can put your answers to questions 3-6 below, or add them directly to the innovations table above next to the relevant innovation (columns 4-7).]

(3) Did the innovations take place in the context of public R&D or private R&D?

(4) Did the innovations originate in the PV industry or somewhere else?

(5) Did some innovations introduce tradeoffs, improving one variable at the expense of another? If so, could you describe the tradeoff?

(6) Time: What is an estimated time range for the innovation?

APPENDIX: INNOVATION EXPLANATIONS

Variables	Innovation number	Innovations	Description
η Module efficiency (unitless)	***Wafer level***		
	1	Electric arc furnace	Produces ~98% pure metallurgical grade-Si (MG-Si) from silica
	2	Siemens process, FBR, UMG-Si	Produces electronic or solar grade silicon (polysilicon)
	3	Czochralski growth for mono-crystalline Si	Produces ingots of Si from feedstock
	4	Multi-crystalline Si casting	Produces ingots of Si from feedstock
	Cell level		
	5	In-line wafer characterization	Testing incoming wafers for cracks and other defects to ensure high quality cells
	6	Wet-chemical etching for texturing	Reducing wire-saw damage, and improving optical performance
	7	Belt furnace, tube diffusion, or ion implantation for P diffusion	Doping and forming pn junction
	8	PECVD or ALD for surface passivation	Depositing layer(s) for surface passivation and antireflective coating
	9	Screenprinting for metal contact formation	Forming metal contacts on the cell to collect the generated current
	Module level		
	10	Reducing variance in cell quality	Output current of cells connected in series is limited by the lowest-performing cell
	11	Tabbing and stringing	Connecting solar cells together
	12	Lamination	Encapsulating solar cells using materials such as glass, EVA to protect them while allowing transmission of light
t Si wafer thickness (μm)	13	Early wafering methods such as inner diameter blade saw	Cutting ingots into wafers
	14	SiC wire sawing	Cutting ingots into wafers -- increased speed compared to inner diameter blade saw. (Thinner and stronger wires are developed over time. Wire diameter, type, and cutting speed determine the material usage.)
	15	Diamond wire sawing	Cutting ingots into wafers
	16	Overcoming limitations in handling and processing thinner wafers	Enabling thinner wafers
U Si utilization (unitless)	17	Wafering methods: wire-sawing, inner diameter blade saw	Cutting ingots into wafers (Wire diameter, type, and cutting speed determine the material usage.)
	18	Production of ingots with larger cross-sectional area	Reducing the fraction of material lost while cropping and shaping the ingot before wafering
p_s Si price ($/kg)	19	Various innovations to tools and materials in MG-Si production and polysilicon production processes	(explained under wafer level innovations improving module efficiency)
y Yield (unitless)	20	Automated transfer machinery	Transfering wafers between manufacturing steps. Automation reduces wafer breakage.
	21	Preventing wafer surface microcracks	Reducing wafer breakage
	22	Reducing the number of and improving the wafer handling steps	Reducing wafer breakage
	23	In-line characterization of wafers	Reducing wafer breakage

	**24**	Laser-based processes as opposed to wet-bench	Reducing wafer breakage
	**25**	Integration of module assembly processes	Simplifying the process, reducing breakage
A **Wafer area (cm2)**	**26**	Better control in Si crystallization	Maintaining material quality across the surface in larger wafers
	**27**	Overcoming limitations in handling large wafers	Allowing increased wafer area while maintaining higher yield
	**28**	Overcoming limitations due to higher series resistance in larger wafers	Allowing increased wafer area while maintaining higher efficiency
α **Packing density (unitless)**	**29**	Quasisquare and square shapes of multicystalline wafers	Allowing more densely-packed modules compared to those using round monocrystalline wafers
c **Non-Si materials costs ($/Wp per wafer area)**	**30**	Development and use of wafer materials such as crucible, argon	Forming ingots with better control e.g. of the oxygen and carbon content
	**31**	Wire slurry, wires, coolant, fixturing for wire-sawing	Slicing ingots into wafers. Wires are used for cutting the ingots into wafers, and slurry facilitates the process.
	**32**	Silver paste (screenprinted to form front contacts)	Collecting the generated current through contacts. Silver paste allows less doping with less capital intensive equipment, and increased throughput.
	**33**	Aluminum (screenprinted on back surface)	Creating back surface field architecture
	**34**	Development and use of other cell materials such as chemicals, screens	Enabling various cell manufacturing processes
	**35**	Development and use of module materials such as encapsulants (e.g.EVA), adhesives, backsheet, tempered low-iron antireflective glass, Al frame, silicone sealant, J-box	Protecting the cells from external impacts, providing support, encapsulating and laminating the connected cells between a Tedlar back and glass front with low-reflectivity allowing transfer of light
K **Plant size (modules/yr or MW/yr)**	**36**	Tools with higher throughput	Increasing output per floor space
	**37**	Turnkey manufacturing facilities	Includes equipment, process technology, and factory control. Readily available
	**38**	Developed equipment manufacturers	Producing one-of-a-kind specialized equipment for PV
	**39**	Developments in material supply industries	Increasing availability and perhaps higher predictability of supply
p0 **Capital, labor, O&M, electricity costs ($/Wp per wafer)**	**40**	Larger silicon ingot sizes	Increasing throughput in Si crystal production
	**41**	Faster processing techniques	Increasing throughput, and decreasing O&M and electricity costs
	**42**	Simplified processes	Increasing throughput, and decreasing O&M and electricity costs
	**43**	Lower-temperature processes	Decreasing O&M and electricity costs
	**44**	Laser-based processes	Decreasing electricity costs
	**45**	Replacing vacuum with non-vacuum processes	Decreasing O&M and electricity costs
	**46**	Increased labor productivity	Decreasing labor costs
	**47**	improvements in manufacturing technologies and equipment	Decreasing capital costs (per W) by increasing throughput, and reduced floor space requirements..
	**48**	Increased automation	Decreasing labor costs, and increasing yield

PV system innovations July 2018

Background. We are evaluating the mechanisms driving photovoltaic (PV) system cost reductions, delving deeply into specific technological innovations that have occurred in the past, and the policies that have encouraged them. This project has three main objectives: (1) identify mechanisms of cost reductions in PV systems (both 'low-level' (e.g. conversion efficiency improvement) and 'high-level' (e.g. R&D efforts)); (2) identify specific innovations that enabled the low-level mechanisms of cost reduction; (3) understand how public policies supporting PV deployment and R&D contributed to PV's cost improvement. Currently we are focusing on objective 2. Our immediate goals are to identify the innovations that were most instrumental in reducing costs since 1980, and better understand how these innovations affected costs. The project as a whole examines both module and balance-of-system (BOS) cost changes and innovations. In this expert feedback request we focus on BOS.

Your input. Please have a look at the material starting on page 2 about PV BOS costs and innovations. On page 2, we describe the variables that we use in a cost model we have developed for BOS costs. On page 3, we then list these variables in a table together with historical innovations that may have caused each variable to improve. We would appreciate your suggestions on how to improve this table.

In particular, we would appreciate your input on

(1) whether our list covers the most important innovations, historically or on-going;
(2) whether the variables that each innovation affects are identified correctly;
(3) whether the innovation took place in the context of public R&D or private R&D
(4) whether the innovation originated in the PV industry or somewhere else
(5) whether the innovation involved tradeoffs, improving one variable at the expense of another. (For instance, an innovation might be focused on maximizing energy yield but may increase inverter costs in $/W.)

You can respond to questions (1) and (2) on page 5. You can respond to questions (3)-(5) either on page 5 or directly in the innovations table in the empty cells next to each innovation.

Description of innovations table. We focus on innovations that may have affected BOS costs over the period 1980-2017. In the first column we list key variables that determine BOS costs. In the second column we assign a number to each innovation. Feel free to use these numbers to refer to innovations in your answers to our questions on page 5. In the third column we list innovations that may have changed each variable and potentially led to cost reduction. An appendix table on page 6 provides brief additional explanations of the innovations.

BOS COST VARIABLES

Our BOS cost equation describes unsubsidized non-module costs to the system owner in $/Wac at the time of purchase. Incentives and operations and maintenance costs are not included.

The BOS equation also includes module-related variables (such as conversion efficiency and module area), and we have requested feedback on the innovations affecting these variables in a separate survey. Here we focus on BOS-specific variables.

c_{inv} Inverter costs ($/W)
η_{inv} Inverter efficiency (rated efficiency, unitless)
ϕ_a Aluminum usage (kg/m of aluminum mounting rail)
τ_{sys} System design time (hours/module)
τ_{mech} Mechanical installation time (hours/module)
τ_{el} Electrical installation time (hours/module)
τ_{PII} Permitting, inspection, and interconnection time (hours/system)
$c_{s.chain}$ Supply chain costs ($/W)

INNOVATIONS TABLE

Variables	Innovation number	Innovations	Origins: Did the innovation take place in the context of public R&D (universities, national labs) or private R&D (industry)?	Origins: Was this innovation specific to PV, or did it come from another industry?	Tradeoffs: Did this innovation introduce any tradeoffs or penalties? If so what were they? (e.g. increasing energy yield, thereby reducing costs per kWh, but increasing inverter costs.)
**c_{inv}, η_{inv} Inverter costs ($/Wac), Inverter efficiency (unitless)**	1	Maximum power point tracking (MPPT)			
	2	Silicon insulated-gate bipolar transistors (Si IGBTs)			
	3	Silicon carbide (SiC) field effect transistors			
	4	Gallium nitride (GaN) field effect transistors			
	5	Thermal management strategies			
	6	Transformerless inverters			
	7	Automated optical inspection procedures			
	8	Soldering machines			
	…	Other innovations?			
**ϕ_a Aluminum usage for mounting system (kg/m)**	9	Wind tunnel testing of mounting systems			
	…	Other innovations?			
**τ_{sys} System design time (hours/ module)**	10	Bid preparation software			
	11	Remote shading analysis software			
	12	Building-integrated PV installation			
	13	Simplified zoning/planning laws			
	14	Prequalification criteria			
	15	AC modules			
	…	Other innovations?			
**τ_{mec} Mechanical installation time (hours/ module)**	16	Integrated mounting systems. ("plug-and-play")			
	17	Railless mounting systems			
	18	Integrated hook/ clamp solutions			
	19	Building-integrated PV installation			
	…	Other innovations?			

Variables	Innovation number	Innovations	Origins: Did the innovation take place in the context of public R&D (universities, national labs) or private R&D (industry)?	Origins: Was this innovation specific to PV, or did it come from another industry?	Tradeoffs: Did this innovation introduce any tradeoffs or penalties? If so what were they?
**τ_el** **Electrical installation time (hours/ module)**	**20**	Microinverters			
	**21**	AC modules			
	**22**	Easy-to-separate PV cables			
	**23**	Y-connectors			
	**24**	Plug-and-play power stations			
	**…**	Other innovations?			
**τ_PII** **Permitting, inspection,and interconnection time (hours/ system)**	**25**	Full online permitting			
	**26**	Online interconnection application and submission			
	**27**	Autopopulating online forms			
	**28**	Template for single line diagram			
	**29**	Cross-training programs for permit staff			
	**30**	Automated engineering review			
	**31**	Solar permit application checklist			
	**32**	Software applications to improve interconnection workflow management for utility			
	**33**	Fast track permitting			
	**…**	Other innovations?			
**c_s.chain** **Supply chain costs ($/Wac)**	**34**	E-commerce marketplaces			
	…	Other innovations?			

4

QUESTIONS

(1) Does our list cover the most important innovations, historically or on-going?

(2) Did we correctly identify the variables that each innovation affects? If not, which revisions would you suggest?

[You can put your answers to questions 3-5 below, or add them directly to the innovations table above next to the relevant innovation (columns 4-6).]

(3) Did the innovations take place in the context of public R&D or private R&D?

(4) Did the innovations originate in the PV industry or somewhere else?

(5) Did some innovations introduce tradeoffs, e.g. by improving one variable at the expense of another? If so, could you describe the tradeoff?

APPENDIX: INNOVATION EXPLANATIONS

Variables	Innovation number	Innovations	Explanation
c_inv, η_inv<br><br>Inverter costs ($/Wac), Inverter efficiency (unitless)	1	Maximum power point tracking (MPPT)	Electric circuit designs and control strategies that adapt inverter resistance to maximize efficiency of power extraction from PV array (i.e. MPPT is the electronic equivalent to a tracker)
	2	Silicon insulated-gate bipolar transistors (Si IGBTs)	Semiconductor switching devices with increased switching frequencies
	3	Silicon carbide (SiC) field effect transistors	Reduced losses (due to wide-bandgap material) allow for higher switching frequencies compared to Si IGBTs, which reduces the need for passive components like coils and capacitors, or allows smaller components. These effects reduce raw material usage and thus costs
	4	Gallium nitride (GaN) field effect transistors	Similar cost-reducing mechanisms as SiC devices; additional advantage is lateral structure which reduces stray inductances and parasitic resistances, which simplifies component packaging
	5	Thermal management strategies	Improved component layouts to increase heat dissipation into environment. Air cooling
	6	Transformerless inverters	Change in national electric code in 2010 that allowed transformerless inverters, which require less raw material (electronic instead of mechanical switching) and are less costly
	7	Automated optical inspection procedures	Automated circuit board inspection after every manufacturing step. Machine compares photograph of circuit board to reference data
	8	Soldering machines	Circuit board is moved through liquid solder paste to bond wire connections to circuit board
ϕ_a<br>Aluminum usage for mounting system (kg/m)	9	Wind tunnel testing of mounting systems	Experimental testing of structural stability of installer equipment allows for novel, reduced-material designs compared to previous, more conservative building codes and standards
τ_sys<br>System design time (h/module)	10	Bid preparation software	Automated design of engineering and sales proposal, including financial analysis, system layout diagram, single-line drawing
	11	Remote shading analysis software	Use of satellite image (instead of on-site measurements) to create 2D image of building. Use of algorithm to construct 3D model and simulate shading. The result is a heat map of site-specific, shading-adjusted irradiance values
	12	Building-integrated PV installation	PV integrated into building design from the beginning of design process to reduce time needed for PV-specific adjustments
	13	Simplified zoning and planning laws	Simplify design requirements, thereby reducing design time
	14	Prequalification criteria	List of criteria for buildings and PV systems that do not require a licensed engineer or achitect to be added to roof structure
	15	AC modules	Eliminating DC circuit reduces design time
τ_mech<br>Mechanical installation time (h/module)	16	Integrated mounting systems ("plug-and-play", "solar platforms")	Prefabricated mounting systems reduce component count and need for tools; fewer and simpler steps reduce installation time
	17	Railless mounting systems	Modules already include rails, can be mounted directly to the roof
	18	Integrated hook and clamp solutions	Integrate standard grounding features in clamp. No need to install grounding separately
	19	Building-integrated PV installation	PV integrated into building design from the beginning of design process to reduce time needed for PV-specific installation

Variables	Innovation number	Innovations	Explanation
τ_el Electrical installation time (h/module)	20	Microinverters	Simpler, faster installation. No extra installation for rapid shutdown requirement established by national electric code (NEC)
	21	AC modules	Simpler, faster installation because microinverter already integrated into module. No extra installation for NEC rapid shutdown requirement (see above)
	22	Easy-to-separate PV cables	Faster, safer installation because co-extruded cables can be separated using fingers instead of cutter. Positive and negative conductor can nevertheless be transported on single spool
	23	Y-connectors	Connectors with one input and two outputs allow simplified ("ready-to-plug") parallel circuit connections
	24	Plug-and-play power stations	Pre-configured electrical connections that require no manual field wiring and reduce overall number of connections that need to be made on-site
τ_PII Permitting, inspection, and interconnection time (h/system)	25	Full online permitting	Enables completion of all aspects of the permit process (application submittal, plan review, fee payment, delivery of approved permits via email or a website) online, often within a short period of time.
	26	Online interconnection application and submission	One single point of entry for applications that previously came via mail, email, fax
	27	Autopopulating online forms	Interconnection and permit application forms that autocomplete based on text recognition
	28	Template for single line diagram	Template for single line diagram that replaced customized single line diagrams
	29	Cross-training programs for permit staff	Cross-training programs for electrical and building inspectors (goal: one site visit instead of two)
	30	Automated engineering review	Automated screening system aggregates equipment information and site specifications provided via application portal, distribution-feeder information, and billing information. This information is then linked to built-in calculations to automatically complete initial review screens (e.g. whether interconnection will exceed acceptable transformer loads)
	31	Solar permit application checklist	Compact summary of technical requirements for homeowners (the innovation is to translate experiences from previous questions into effective information)
	32	Software applications to improve interconnection workflow management for utility	Software that consolidates internal management and processing of interconnections under different interconnection rules. Simplifies document retention and retrieval by enabling departments within a company to interact with a common database
	33	Fast track permitting	Expedited permitting for small-scale, standard systems
c_s.chain Supply chain costs ($/Wac)	34	E-commerce marketplaces	Enables smaller firms to gain centralized access to a larger pool of products. Could in future allow aggregating orders by multiple small installers to benefit from bulk purchase prices

References

1. Kavlak G, McNerney J, Trancik JE. Evaluating the causes of cost reduction in photovoltaic modules. Energy Policy. 2018;123:700 – 710.

2. Goodrich A, James T, Woodhouse M. A wafer-based monocrystalline silicon photovoltaics road map: Utilizing known technology improvement opportunities for further reductions in manufacturing costs. Solar Energy Materials and Solar Cells. 2013;114:110–135.

3. Luque A. Photovoltaics in 1986: Routes to Low Cost. In: Goetzberger A, Palz W, Willeke G, editors. Seventh E.C. Photovoltaic Solar Energy Conference. Dordrecht: Springer Netherlands; 1987. p. 9–18.

4. Goetzberger A, Hebling C, Schock HW. Photovoltaic materials, history, status and outlook. Materials Science and Engineering: R: Reports. 2003;40(1):1–46.

5. Mitchell K. Renaissance of Czochralski silicon photovoltaics. Progress in Photovoltaics: Research and Applications. 1994;2(2):115–120.

6. Goodrich A, James T, Woodhouse M. Solar PV Manufacturing Cost Analysis: U.S. Competitiveness in a Global Industry. NREL Presentation. 2011;NREL/PR-6A20-53938.

7. Woodhouse M, Smith B, Ramdas A, Margolis R. Crystalline Silicon Photovoltaic Module Manufacturing Costs and Sustainable Pricing: 1H 2018 Benchmark and Cost Reduction Roadmap. NREL Technical Report. 2019;NREL/TP-6A20-72134.
